# Mutant p53-ENTPD5 control of the calnexin/calreticulin cycle: a druggable target for inhibiting integrin-α5-driven metastasis

**DOI:** 10.1186/s13046-023-02785-z

**Published:** 2023-08-10

**Authors:** Evangelos Pavlakis, Michelle Neumann, Nastasja Merle, Ronja Wieboldt, Michael Wanzel, Viviane Ponath, Elke Pogge von Strandmann, Sabrina Elmshäuser, Thorsten Stiewe

**Affiliations:** 1 Institute of Molecular Oncology, Philipps-University, 35043 Marburg, Germany; 2 Universities of Giessen and Marburg Lung Center (UGMLC), German Center for Lung Research (DZL), Marburg, 35043 Germany; 3 Institute for Tumor Immunology, Philipps-University, 35043 Marburg, Germany; 4 Genomics Core Facility, Philipps-University, 35043 Marburg, Germany; 5 Institute for Lung Health (ILH), Justus Liebig University, 35392 Giessen, Germany

**Keywords:** p53, Tumor suppressor gene, Metastasis, ENTPD5, Calnexin, Calreticulin, Glycoprotein biosynthesis, Chaperone, Integrin

## Abstract

**Background:**

*TP53*, encoding the tumor suppressor p53, is frequently mutated in various cancers, producing mutant p53 proteins (mutp53) which can exhibit neomorphic, gain-of-function properties. The latter transform p53 into an oncoprotein that promotes metastatic tumor progression via downstream effectors such as ENTPD5, an endoplasmic reticulum UDPase involved in the calnexin/calreticulin cycle of N-glycoprotein biosynthesis. Elucidating the mechanisms underlying the pro-metastatic functions of the mutp53-ENTPD5 axis is crucial for developing targeted therapies for aggressive metastatic cancer.

**Methods:**

We analyzed pancreatic, lung, and breast adenocarcinoma cells with p53 missense mutations to study the impact of mutp53 and ENTPD5 on the N-glycoproteins integrin-α5 (ITGA5) and integrin-β1 (ITGB1), which heterodimerize to form the key fibronectin receptor. We assessed the role of the mutp53-ENTPD5 axis in integrin-dependent tumor-stroma interactions and tumor cell motility using adhesion, migration, and invasion assays, identifying and validating therapeutic intervention targets. We employed an orthotopic xenograft model of pancreatic ductal adenocarcinoma to examine *in viv*o targeting of mutp53-ENTPD5-mediated ITGA5 regulation for cancer therapy.

**Results:**

Mutp53 depletion diminished ITGA5 and ITGB1 expression and impaired tumor cell adhesion, migration, and invasion, rescued by ENTPD5. The mutp53-ENTPD5 axis maintained ITGA5 expression and function via the calnexin/calreticulin cycle. Targeting this axis using ITGA5-blocking antibodies, α-glucosidase inhibitors, or pharmacological degradation of mutp53 by HSP90 inhibitors, such as Ganetespib, effectively inhibited ITGA5-mediated cancer cell motility in vitro. In the orthotopic xenograft model, Ganetespib reduced ITGA5 expression and metastasis in an ENTPD5-dependent manner.

**Conclusions:**

The mutp53-ENTPD5 axis fosters ITGA5 and ITGB1 expression and tumor cell motility through the calnexin/calreticulin cycle, contributing to cancer metastasis. ITGA5-blocking antibodies or α-glucosidase inhibitors target this axis and represent potential therapeutic options worth exploring in preclinical models. The pharmacologic degradation of mutp53 by HSP90 inhibitors effectively blocks ENTPD5-ITGA5-mediated cancer cell motility and metastasis in vivo, warranting further clinical evaluation in p53-mutant cancers. This research underscores the significance of understanding the complex interplay between mutp53, ENTPD5, and the calnexin/calreticulin cycle in integrin-mediated metastatic tumor progression, offering valuable insights for the development of potential therapeutic strategies.

**Supplementary Information:**

The online version contains supplementary material available at 10.1186/s13046-023-02785-z.

## Background

*TP53*, the gene encoding the tumor suppressive transcription factor p53, is the most frequently mutated gene in a wide variety of cancers, with mutations present in approximately 50% of all cancer patients [1]. Unlike other tumor suppressors, *TP53* is predominantly inactivated by missense mutations, resulting in the production of mutant proteins [1–3]. These mutant p53 proteins (mutp53) not only lose their tumor-suppressive functions but also display dominant-negative and neomorphic properties (often referred to as gain-of-function, GOF), switching p53 from a tumor suppressor to a protein with oncogenic activity [3–6]. The oncogenic consequences of *TP53* missense mutations are widely believed to play a key role in multiple steps of tumor initiation, progression, metastasis and treatment response [4, 5]. Among these, the pro-metastatic activity of mutant p53 proteins is arguably the most prominent function, as demonstrated, for example, by the increased metastasis of tumors arising in mutant p53 knock-in compared to p53 knock-out mice [7–10]. Understanding the mechanisms underlying the pro-metastatic functions of *TP53* missense mutations could pave the way for the development of novel diagnostic tools and targeted therapies for patients with aggressive metastatic cancer.

Several mutp53 targeting approaches are currently being evaluated in preclinical models and early clinical trials, including direct targeting of mutp53 itself [11–13]. The activity of wild-type p53 is tightly controlled by E3 ubiquitin ligases, such as Mdm2 and Chip/Stub1, which tag p53 for proteasomal degradation [14]. In contrast, p53 missense mutants are shielded from degradation by heat shock proteins, which are repressed by wild-type p53 and upregulated by *TP53* loss of heterozygosity and oncogenic signals [15, 16]. As a result, missense mutants accumulate in cancer cells, a process considered diagnostic and essential for GOF activities [17]. In preclinical models, targeting the mechanisms that contribute to the stabilization of mutp53 in cancer cells has demonstrated the ability to induce regression of tumors carrying p53 missense mutations [18, 19].

Focusing on downstream effector pathways responsible for specific oncogenic functions, such as metastasis, is another strategy to target mutp53 [11, 12]. Metastasis is a complex process involving the detachment of cells from their primary site, as well as adhesion to and migration along fibers present in the extracellular matrix (ECM). Migrating tumor cells invade through tissue borders to gain access to distant tissues through the vasculature. Mutant p53 directly controls the expression of genes encoding cell surface receptors and secreted factors, and indirectly modulates cellular protein biogenesis and secretion pathways [20–25]. The combined activities of mutp53 shape the interaction and communication between tumor cells and their microenvironment, ultimately contributing to metastatic tumor progression [9, 20, 21].

One mechanism that connects mutp53 to metastasis operates through ENTPD5 (ectonucleoside triphosphate diphosphohydrolase 5), a UDPase located in the endoplasmic reticulum (ER) [25–28]. Research in mice has shown that depleting ENTPD5 or mutp53 reduces breast cancer metastasis to the lung, while enforced ENTPD5 expression rescues lung colonization by cancer cells that have been depleted of mutp53 [25]. This suggests that the mutp53-ENTPD5 axis is necessary and sufficient for mutp53-driven metastasis. ENTPD5 is induced by several p53 mutants in different cancer types through mutp53 recruitment to the ENTPD5 core promoter via the transcription factor Sp1 [25]. ENTPD5 is believed to function in the ER through the calnexin/calreticulin (CANX/CALR) chaperone system [28]. Unfolded N-glycosylated proteins are tagged at the core glycan with a single glucose moiety by UDP-glucose:glycoprotein glucosyltransferase (UGGT), and then seized by CANX/CALR to promote proper folding and prevent the release of improperly folded proteins from the ER [29–32]. ENTPD5 terminates the chaperone cycle by cleaving UDP to UMP, which in turn exits the ER via an antiporter in exchange for new UDP-glucose molecules [28]. The upregulation of the folding capacity by mutp53 is expected to boost the biogenesis of those N-glycoproteins, which are especially reliant on the CANX/CALR cycle for proper folding and functioning [33]. Heavily N-glycosylated proteins are, for example, receptor tyrosine kinases and integrins with established pro-metastatic activity [25–27, 34]. However, the specific N-glycoproteins controlled by the mutp53-ENTPD5 axis and promoting metastasis remain to be identified.

Here, we provide evidence that the mutp53-ENTPD5 axis operates through the CANX/CALR cycle to maintain high expression levels of integrin-α5 (ITGA5), which pairs with integrin-β1 (ITGB1) to form the primary fibronectin receptor. We demonstrate that ITGA5 is required for the pro-invasive and pro-metastatic potential of pancreatic tumor cells with a p53 missense mutation. Our findings strongly emphasize the crucial role of this novel pathway in driving metastatic tumor progression and demonstrate that targeting mutp53 stability, the CANX/CALR cycle, or ITGA5 can effectively interfere with mutp53-mediated pro-metastatic functions. This highlights the importance of the mutp53-ENTPD5 control of ITGA5 as a potential therapeutic target for p53-mutant cancers.

## Materials and methods

### Cell Culture

Pancreatic cancer (PANC-1, MIA PaCa-2), breast cancer (MDA-MB-231), lung cancer (PC-9, H1975, H1299), and HEK293T cell lines were obtained from the American Type Culture Collection (ATCC) through their European distributor LGC Standards GmbH and cultured in Dulbecco's Modified Eagle's Medium (DMEM) supplemented with 10% fetal bovine serum (FBS), 1% (v/v) Penicillin (10.000 U mL^−1^)/Streptomycin (10 mg mL^−1^), and 0.4% Amphotericin B (250 μg mL^−1^). MIA PaCa-2 cells with tet-inducible expression of ENTPD5, p53^R248W^, or p53^R175H^ have been described [25] and were generated by lentiviral transduction using the pInducer20 system [35]. Ovarian carcinoma cell lines (OC-121, OC-58; [36]) were cultured in a 1:1 mix of DMEM/Ham’s F12 + 2 mM stabilized Glutamine (Millipore/Biochrome) and M199 medium with the addition of 5% FCS (Fetal Calf Serum), 20 μg mL^−1^ Insulin solution from bovine pancreas, 10 ng mL^−1^ hrEGF (human recombinant epidermal growth factor), 500 ng mL^−1^ Hydrocortisone, 25 ng mL^−1^ Cholera toxin, 10 mM HEPES (4-(2-hydroxyethyl)-1-piperazineethanesulfonic acid), 10 μg mL^−1^ Transferrin, 0.2 pg mL^−1^ Triiodothyronine, 5 μg mL^−1^ O-phosphoryl ethanolamine, 8 ng mL^−1^ selenious acid, 25 ng mL^−1^ all-trans retinoic acid, and 5 μg mL^−1^ linoleic acid. Cells were maintained at 37 °C with 5% CO_2_ in a humid atmosphere (95%).

The following reagents were used for cell cultures and treatments at the indicated dose ranges: Suberoylanilide hydroxamic acid (SAHA, SML0061, Sigma-Aldrich; 10–15 µM), Doxycycline (D9891, Sigma-Aldrich; 1 µg mL^−1^), Acarbose (A8980, Sigma-Aldrich; 1–6 µM), Nutlin-3a (SML0580, Sigma-Aldrich; 10 µM), 17-Allylamino-17-demethoxygeldanamycin (17-AAG, Tanespimycin, S1141, Selleckchem; 10–15 µM), Ganetespib (S1159, Selleckchem; 0.5–1 µM), UV-4 (HY-U00160, MedChemExpress; 5–20 µM), anti-Integrin α5 Antibody (clone P1D6, MAB1956Z, Merck; 15 µg cm^−2^), Fibronectin from bovine plasma (F1141, Sigma-Aldrich; 5–10 µg mL^−1^), Collagen I from rat tail (A1048301, Thermo Fisher Scientific; 12 µg cm^−2^).

For retroviral transduction of cells, Platinum-E cells (Cell Biolabs) were transfected with pMSCV-Firefly-T2A-Gaussia plasmids using a standard calcium phosphate protocol [37, 38]. After three days, retrovirus-containing supernatants were collected, filtered through a 0.45 μm filter, and supplemented with 8 μg mL^−1^ polybrene for infection. MIA PaCa-2 cells were infected in 6-well plates (Sarstedt) using spinoculation for one hour at 600 g and 37 °C.

### RNAi experiments

Cell cultures were transfected with siRNAs using Lipofectamine RNAiMax (#13,778,075, Thermo Fisher Scientific) at a final concentration of 20 nM, unless otherwise stated, following the manufacturer’s protocol. Cells were harvested 72 h after transfection. The siRNAs used were obtained from Dharmacon with ON-TARGET plus modification, unless otherwise stated: nsi153 (non-targeting siRNA pool of the following four siRNAs): nsi1 (UGG UUU ACA UGU CGA CUA A), nsi2 (UGG UUU ACA UGU UGU GUG A), nsi3 (UGG UUU ACA UGU UUU CUG A), nsi4 (UGG UUU ACA UGU UUU CCU A); p53-si1 (GAC UCC AGU GGU AAU CUA C), p53-si3 (GCA GUC AGA UCC UAG CGU C), p53-si4 (GGA CAU ACC AGC UUA GAU UUU, siGenome); ENTPD5-si6 (AGA CUU GGU UUG AGG GUA U), ENTPD5-si7 (CAG GAC AGC UUC CAA UUC U), ENTPD5-si8 (CAU AUU AGC UUG GGU UAC U), ENTPD5-si9 (CGA GAU GGU UGG AAG CAG A); CANX-si8 (UGA CAU GAC UCC UCC UGU A); CANX-si9 (GAA AGA CGA UAC CGA UGA U); CALR-si7 (GCA CGG AGA CUC AGA AUA C); CALR-si8 (GAA GCU GUU UCC UAA UAG U); UGGT-si5 (GAG CUG ACA UUG CGG AGU U); UGGT-si8 (GGG ACG CUC UGA AGA UAU U); ITGA5-si10 (ACA CGU UGC UGA CUC CAU U); ITGA5-si11 (CAA ACG CUC CCU CCC AUA U); ITGB1-si5 (GUG CAG AGC CUU CAA UAA A); ITGB1-si8 (GGG CAA ACG UGU GAG AUG U).

### RTqPCR

RNA isolation from cell cultures was performed using the RNeasy Mini kit (Qiagen) following the manufacturer's instructions. cDNA synthesis was done using the SuperScript VILO cDNA Synthesis kit (Thermo Fisher Scientific) following the manufacturer's instructions. Real-time quantitative PCR (RT-qPCR) was performed using ABsolute QPCR Mix with SYBR Green (Thermo Fisher Scientific) and a LightCycler 480 (Roche). The oligos used for RT-qPCR were designed using Primer3 software, and their specificity was verified using BLAST searches against the NCBI database. The oligo sequences used were as follows: ITGA5_for 5’-TGC AGT GTG AGG CTG TGT ACA-3’; ITGA5_rev 5’-GTG GCC ACC TGA CGC TCT-3’; GAPDH_for 5’-CTA TAA ATT GAG CCC GCA GCC-3’; GAPDH-rev 5’-ACC AAA TCC GTT GAC TCC GA-3’.

### Western blot

Cells were harvested and lysed in NP-40 lysis buffer (50 mM Tris–HCl pH 7.4, 150 mM NaCl, 5 mM EDTA pH 8.0, 2% NP-40) containing protease inhibitors (cOmplete ULTRA tablets mini, Roche) and subjected to Western blotting as previously described [25]. Briefly, protein samples were separated by SDS-PAGE and transferred to nitrocellulose membranes. The membranes were then incubated with primary antibodies, including mouse anti-Integrin-α5 (C-9, SC-376199, Santa Cruz Biotechnology; 1:200), mouse anti-Integrin-β1 (JB1B, sc-59829, Santa Cruz Biotechnology; 1:200), mouse anti-p53 (DO-1, SC-126, Santa Cruz Biotechnology; 1:10,000), rabbit anti-ENTPD5 (EPR3783, ab92542, Abcam; 1:2,500), rabbit anti-CANX (ab22595, Abcam; 1:1000), rabbit anti-CALR (ab92516, Abcam; 1:1000), mouse anti-UGGT1 (H-9, sc-374565, Santa Cruz Biotechnology; 1:200), and mouse anti-β-actin (AC-15, ab6276, Abcam; 1:5,000). The secondary antibodies used were mouse IgG HRP-linked (NA9310; 1:10,000) and rabbit IgG HRP-linked (NA9340; 1:10,000) from Amersham.

### Immunohistochemistry and confocal immunofluorescence

Tissues were fixed in buffered formalin and embedded in paraffin. The embedded tissues were cut into sections and fixed on glass slides overnight at 37 °C. Staining was performed as previously described [39]. Briefly, the slides were deparaffinized, rehydrated, and subjected to antigen retrieval with EDTA (pH 8.0) for ENTPD5, Universal HIER (Abcam; ab208572) for Integrin-α5, and Citrate (pH 6.0) for p53. Primary antibody incubation was performed using mouse anti-Integrin-α5 (C-9; SC-376199; Santa Cruz Biotechnology; 1:200), mouse anti-p53 (DO-1; sc-126; Santa Cruz Biotechnology; 1:1,000), and rabbit anti-ENTPD5 (EPR3784; ab108603; Abcam; 1:400). Images were acquired using the Leica Aperio Versa slide-scanner and Leica Aperio eSlide Manager software v. 1.0.3.37.

For confocal immunofluorescence microscopy, MIA PaCa-2 cells were seeded at a density of 2,500 cells per well in a 96-well plate (µ-Plate 96 Well Black ibiTreat, #89,626, ibidi GmbH). After 3 days, the cells were fixed with ice-cold methanol for 10 min and permeabilized with 0.1% NP-40/PBS (two times for 5 min) at RT. Following a 45 min blocking step at 37 °C with 5% FCS/0.1% NP-40/PBS, the cells were incubated with a mixture of anti-PDIA3 (AMAB 90988, Merck, 1:500) and anti-ENTPD5 (EPR3784, ab108603, Abcam, 1:500) antibodies at 37 °C for 45 min. Subsequently, a secondary antibody mixture (donkey anti-rabbit ready probes Alexa Fluor 488; R37118, Invitrogen, 1:400; goat anti-mouse Alexa Fluor Plus 555, A32727, 15,698,545, Invitrogen, 1:400) along with DAPI (100 nM) were added to the cells for an additional 45 min incubation at 37 °C. The fluorescence signal was measured using a Leica SP8 confocal microscope equipped with a 63 × objective (1024 × 1024 pixels) and analyzed using the LAS X software (Leica Microsystems). Colocalization of ENTPD5 and PDIA3 was analyzed with the Coloc 2 plug-in (release 3.0.6) for ImageJ (version 2.9.0, distribution by Fiji) using the pixel intensity Pearson correlation over space method.

### Proliferation assay

Tumor cell proliferation was monitored in real-time using an IncuCyte S3 Live-Cell Analysis System (Sartorius). Cells were seeded in 96-well plates overnight and, depending on the experiment, transfected with siRNAs or treated with compounds. At least two phase-contrast images per well were captured every two hours at 10 × magnification, with three replicate wells per treatment condition. Confluence analysis was performed with IncuCyte S3 2018A software in Phase Object Confluence mode, using a segmentation score of 0.7 and excluding objects smaller than 500 μm^2^. Confluence curves were normalized to the confluence of non-transfected or non-treated reference cells at the end of the time course. Proliferation was measured as the area under the confluence curve (AUC) using GraphPad Prism (version 9.4.1).

### Adhesion assays

Cells were transfected in a 6-well plate format and harvested with Accutase (Sigma-Aldrich) in DMEM after 72 h. Each 6-well plate was split into multiple 96-well plates of a low-binding dish (Sarstedt) that were pre-coated overnight at 37 °C with 5 μg/cm^2^ Fibronectin. The cells were allowed to adhere for a designated period of time, and non-attached cells were removed by washing twice with 1 × PBS. The attached cells were then measured using the Cell Titer Glo Assay (Promega) according to the manufacturer’s protocol, in an Orion II Microplate Luminometer (Berthold Detection Systems GmbH). To ensure that the measured effect was due to gene knockdown and not a lower cell number, non-coated 96-well plates containing the suspension of the total number of cells seeded for each condition were used as seeding controls for normalization.

### Cell spreading assays

MIA PaCa-2 cells were stably transduced with pWXL-sAC-GFP lentivirus, expressing a nucleocytoplasmic shuttling Actin-Chromobody-TagGFP [40], and enriched for homogenous GFP expression by FACS. MIA PaCa-2-sAC-GFP cells were transfected with siRNAs and incubated for 72 h. Spreading was analyzed on 8-well μ-slides (ibiTreat) pre-coated overnight at 37 °C with 10 μg mL^−1^ fibronectin (FN) in PBS. Coating fluids were discarded and 30,000 cells in 200 μL medium were seeded per 8-well chamber. Spreading was monitored in real-time using spinning disc microscopy (Spinning Disc Axio Observer Z1, Zeiss). Spreading of the cells was assessed and discriminated from non-spreading cells by z-stack position and cell shape.

### Migration and invasion assays

To prepare for migration and invasion assays, MIA PaCa-2 cells (9 × 10^5^) were transfected with 10 nM siRNA in a 6-well plate format. In experiments, where doxycycline was used to induce ENTPD5 expression, it was added to the media 24 h after transfection. 72 h after transfection, the cells were dissociated with Accutase (Sigma-Aldrich), counted using a Beckman Coulter, and harvested.

For migration assays, 9 × 10^5^ cells were seeded onto the top chamber of 24-well transwell inserts (Sarstedt) that had been pre-coated overnight with 100 µL of 5 μg cm^−2^ FN. For invasion assays, transwells were coated overnight with a gel-matrix composed of 12 μg cm^−2^ Collagen I and 10 μg cm^−2^ FN, in a final volume of 100 µL. Transwells without coating served as controls for migration assays, and transwells coated only with Collagen I served as controls for invasion assays. In parallel, 96-well plates were seeded with the same number of cells and used to control and normalize for differences in the number of seeded cells. After seeding the cells on the transwells, they were allowed to adhere for 1–2 h before the lower chamber was filled with 500 µL DMEM containing 10% FBS, and the upper chamber with 300 µL DMEM with 0.5% FBS. In the case of drug treatments, media (in both top and lower chambers) were supplemented with the specific compounds. Migration assays were stopped 48 h after seeding, invasion assays 72 h after seeding.

The cells that remained in the top chamber were removed by thorough washing with 1 × PBS and wiping with cotton swabs. The cells that passed through the transwell membrane and resided on the bottom surface of the transwell or in the lower chamber were quantified using one of two methods: (1) Cells were dissociated using Accutase, harvested, and measured on a plate reader luminometer (ORION II, Titertek-Berthold) using the Cell Titer Glo Assay (Promega), following the manufacturer’s protocol. (2) Alternatively, cells were fixed with 70% ethanol, stained with 0.2% crystal violet in 10% ethanol, and photographed. The stained cells were then destained in 20% acetic acid for 10 min, and the absorbance of the collected solution was measured at 590 nm on a plate reader (BioTek Synergy HT). In all assays, the measured number of cells that had passed through the transwell membrane was normalized to the reference cells and the seeding controls.

### Experimental in vivo metastasis model

Animal experiments were conducted in compliance with the German animal welfare law and the European legislation for the protection of animals used for scientific purposes (2010/63/EU), and were approved by the regional board (RP Giessen). MIA PaCa-2 cells, with and without tet-inducible ENTPD5 expression, were ex vivo labeled with *Gaussia princeps* (GLuc) and firefly luciferases (FLuc) by retroviral transduction. 1 × 10^6^ tumor cells were mixed with 20 μL growth factor-reduced Matrigel (Corning) and orthotopically injected into the pancreata of male and female *Rag2*^tm1.1Flv^;*Il2rg*^tm1.1Flv^ immunocompromised mice aged 3–6 months. Mice were kept under specified pathogen-free (SPF) conditions in individually ventilated cages with a 12–12 h light–dark cycle and a standard Altromin housing diet. The orthotopic pancreatic model was described in detail in previous studies [41–43]. Briefly, mice were anesthetized using 50 µg/kg Fentanyl (Hameln-Pharma), 500 µg/kg Medetomidine (Alfavet), and 5,000 µg/kg Midazolam (Hameln-Pharma). The abdominal area was shaved and disinfected with Povidone-iodine (B. Braun), after which the left side of the abdominal cavity was opened, and the spleen and pancreas were extracted and injected with a 20 µL cell suspension. After approximately 1 min, the spleen and pancreas were returned to the abdominal cavity, and muscle and skin were closed with 6.0 threads. Mice were administered 1,200 µg/kg Naloxone (Inresa Arzneimittel), 1200–1500 µg/kg Atipamezole (Zoetis), and 500 µg/kg Flumazenil (Inresa Arzneimittel) to reverse anesthesia, and received Meloxicam (CP Pharma or Böhringer Ingelheim) for a minimum of 3 days. To induce ENTPD5 expression in tumors with tet-inducible ENTPD5, the mice were provided with drinking water containing 1 mg mL^−1^ doxycycline and 2% (w/v) sucrose, in darkened bottles, starting 3–4 days before surgery. The drinking water was renewed twice a week with freshly prepared doxycycline. Ganetespib (75 mg kg^−1^ body weight; STA-9090, Adoq Bioscience LLC) or vehicle was administered once per week starting 2–3 days before surgery by intravenous tail vein injection. Blood samples were taken once a week starting 1–2 days before surgery to monitor tumor growth based on GLuc blood levels, as previously described [44, 45]. Four weeks post-surgery, the mice were euthanized and their pancreata and livers (primary metastatic organs) were collected. The pancreata were processed for immunohistochemical analyses, and the livers were lysed using a TissueLyser LT (Qiagen) and the Luciferase cell culture lysis 5 × reagent (Promega), according to the manufacturer’s protocol. Firefly luciferase activity was quantified in the liver lysates using the Beetle-Juice Luciferase assay Firefly (PJK) on a plate reader luminometer (ORION II, Titertek-Berthold), following the manufacturer’s protocol.

### Survival analysis

The correlation between ITGA5 and ITGB1 expression based on TCGA and GTEx RNAseq data and survival of cancer patients was analyzed using the Gene expression Profiling Interactive Analysis 2 (GEPI A2) online platform (http://gepia2.cancer-pku.cn) [46]. Using the Survival Analysis interface, we analyzed the pancreatic adenocarcinoma (PAAD), breast cancer (BRCA), lung adenocarcinoma (LUAD), and ovarian cancer (OV) cohorts for disease-free survival using the quartile cutoff with default parameters.

### Statistical analyses

The plots and statistical analyses in this study were created using GraphPad Prism (version 9.4.1). ITGA5 protein levels were quantified from Western blots using ImageJ software (version 1.51). Graphics were assembled in Adobe Illustrator (version 26.5.2) and BioRender.com. The results presented in the graphs represent the mean or median values obtained from n replicates. The error bars in the figures indicate the standard deviation (SD) unless stated otherwise. To compare multiple groups, a one-way ANOVA was used in combination with a multiple comparisons test. For three or more groups that have been divided into two independent variables (such as treatment and genotype), a two-way ANOVA was used in combination with a multiple comparisons test. The ANOVA results and selected pairwise comparisons are reported in the figures. A p-value less than 0.05 was considered statistically significant.

## Results

### Integrin-α5/β1 expression is dependent on mutp53/ENTPD5 in different cancer entities

**Fig. 1:**
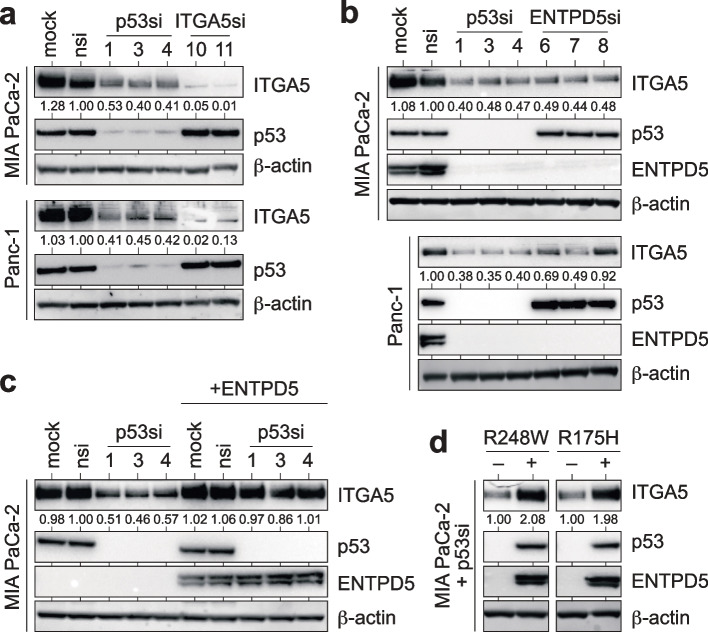
Mutant p53 controls ITGA5 expression via ENTPD5. **a-b** Western blots of pancreatic ductal adenocarcinoma cell lines (MIA PaCa-2, Panc-1) for expression of p53, ENTPD5, and ITGA5 following transfection with siRNAs targeting p53, ENTPD5 or ITGA5 as indicated. **c** Western blot for p53, ITGA5, and ENTPD5 following p53-depletion in MIA PaCa-2 cells with doxycycline-inducible ENTPD5 expression. Doxycycline was added to the media 24 h before siRNA transfection. **d** Western blot for p53, ITGA5, and ENTPD5 following p53 depletion in MIA PaCa-2 cells with doxycycline-inducible expression of p53^R248W^ or p53^R175H^ mutants. Doxycycline was added 24 h before transfection with an siRNA targeting the 3’UTR of the endogenous mutp53. In all Western blots, ITGA5 levels were quantified by ImageJ and normalized to the nsi control. β-actin served as a loading control. Mock: non-transfected cells; nsi: non-targeting control siRNA

Integrins are a family of heterodimeric transmembrane receptors consisting of alpha and beta subunits that play a critical role in cell–cell and cell-ECM interactions and are fundamental for tumor cell migration and invasion [47]. As the integrins ITGA5 and ITGB1 are among the most heavily N-glycosylated integrins [34] and depend on the CANX/CALR cycle for proper folding [48], we investigated whether the mutp53-ENTPD5 axis affects their expression. Our results show that mutp53 depletion strongly downregulates ITGA5 expression in tumor cell lines from pancreatic (MIA PaCa-2, Panc-1), breast (MDA-MB231), and lung (PC9, H1975) adenocarcinomas, but not in two p53 mutant ovarian carcinoma cell lines (Fig. 1a and Supplemental Fig. S1a-e). In parallel, we observed downregulation of ENTPD5 upon mutp53 depletion, consistent with previous reports [25]. To investigate the dependence of ITGA5 expression on ENTPD5, we transiently depleted ENTPD5 and observed a reduction in ITGA5 protein expression similar to mutp53-depleted samples (Fig. 1b and Supplemental Fig. S1a-c). Notably, mutp53 or ENTPD5 depletion had no effect on *ITGA5* mRNA expression, indicating that ITGA5 levels are not transcriptionally regulated by the mutp53-ENTPD5 axis (Supplemental Fig. S1f). Depletion of mutant p53 or ENTPD5 also diminished the expression of ITGB1, the obligate binding partner of ITGA5, in the pancreatic (MIA PaCa-2), breast (MDA-MB-231), and lung adenocarcinoma (H1975), but not in the ovarian carcinoma cells (Supplemental Fig. 1g-j). These results suggest that the high-level expression of ITGA5 and ITGB1 in p53-mutant cancer cells requires both mutp53 and ENTPD5.

Expression of ITGA5 and ITGB1 inversely correlated with disease-free survival in pancreatic, but not breast, lung, or ovarian cancer (Supplemental Fig. S2). Therefore, we decided to focus our further studies on pancreatic cancer. Re-expression of ENTPD5 in mutp53-depleted MIA PaCa-2 cells restored ITGA5 protein levels, indicating that mutp53 and ENTPD5 have an epistatic effect in maintaining high-level ITGA5 expression (Fig. 1c). The mutp53-dependent ITGA5 expression was observed in cell lines with different p53 missense mutations (R248W in MIA PaCa-2, R248Q in PC9, R273H in Panc-1 and H1975, R280K in MDA-MB-231) (Fig. 1a-c and Supplemental Fig. 1a-c). Additionally, we found that ITGA5 protein levels were rescued in mutp53-depleted MIA PaCa-2 cells not only by ectopic expression of the endogenous R248W mutation but also by R175H (Fig. 1d), underlining that the mutp53-ENTPD5-dependent expression of ITGA5 is not limited to a specific *TP53* missense mutation. Overall, our findings indicate that mutant p53 upregulates the protein levels of ITGA5 and ITGB1 through ENTPD5. Furthermore, this regulatory mechanism is not limited to a specific cancer type and is observed across various missense mutants of p53.

### ENTPD5 is required for ITGA5-mediated tumor cell adhesion, migration, and invasion

**Fig. 2:**
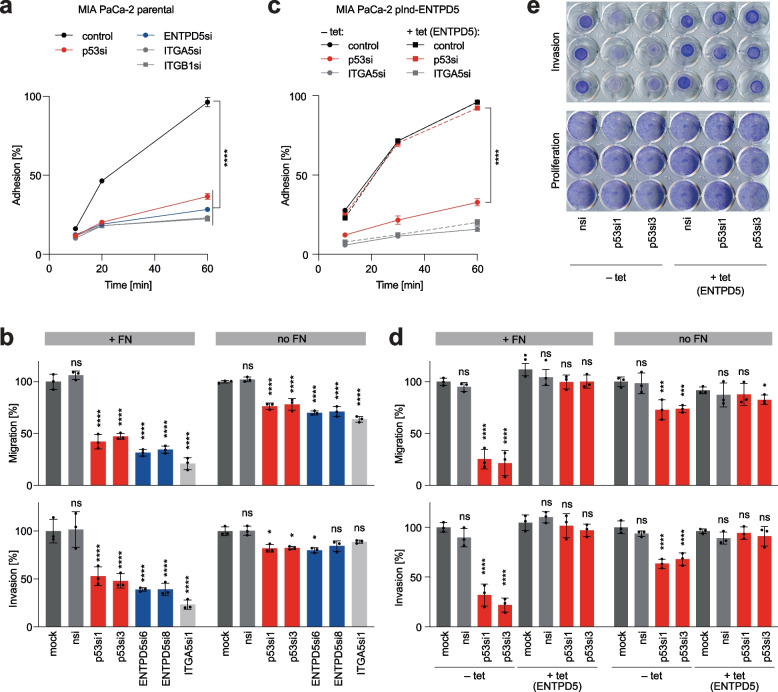
Mutant p53 signaling via ENTPD5 is required for FN-mediated cell adhesion, migration, and invasion. **a** Adhesion kinetics of MIA PaCa-2 cells to fibronectin (FN) following siRNA-mediated depletion of p53, ENTPD5, ITGA5 or ITGB1. Adhesion is expressed as the percentage of the seeded cell number. **b** Transwell migration and invasion of MIA PaCa-2 cells depleted of p53, ENTPD5, or ITGA5, assayed in the presence or absence of FN. Numbers of migrated and invaded cells were normalized to seeding controls and expressed as the percentage of mock-treated cells. **c** Adhesion kinetics of MIA PaCa-2 pIND-ENTPD5 cells (with tet-inducible expression of ENTPD5) to FN. Cells were induced with doxycycline (+ tet), transfected with siRNAs as indicated and analyzed as in **a**. **d-e** Transwell migration and invasion of MIA PaCa-2 pIND-ENTPD5 cells in the presence or absence of FN. Cells were induced with doxycycline (+ tet) as indicated, transfected with siRNAs, and analyzed as in **b**. **d** Quantification of assay results. **e** Representative images of crystal violet-stained transwell inserts containing FN. Proliferation control wells document comparable cell seeding and viability. All graphs show the mean ± SD of *n* = 3 independent experiments. Significance was tested by two-way ANOVA followed by Dunnett’s multiple comparisons test: *, *p* < 0.05; **, *p* < 0.01; ***, *p* < 0.001; ****, *p* < 0.0001; ns, not significant

ITGA5, in complex with ITGB1, forms the primary receptor for fibronectin (FN), which mediates cellular interactions with FN fibers in the extracellular matrix [47]. To explore the contribution of the mutp53-ENTPD5 axis to ITGA5 and ITGB1 activity, we conducted experiments to measure cellular adhesion to FN. We observed significantly reduced adhesion of MIA PaCa-2 cells to FN-coated plates upon depletion of either ITGA5 or ITGB1 (Fig. 2a). This demonstrated that FN-binding is dependent on ITGA5 and ITGB1 and confirmed that the assay correctly measured the FN-binding activity of ITGA5 and ITGB1. Importantly, consistent with the role of mutp53 and ENTPD5 in controlling ITGA5 and ITGB1 protein expression, we observed a similarly reduced adhesion when mutp53 or ENTPD5 were depleted (Fig. 2a). Adhesion of MDA-MB-231 breast and H1975 lung adenocarcinoma cells was likewise reduced by depletion of mutp53, ENTPD5, ITGA5, or ITGB1 (Supplemental Fig. S3a-b). In the ovarian carcinoma cells, where ITGA5 and ITGB1 were expressed independently of mutp53 and ENTPD5, adhesion to FN was only reduced by direct depletion of ITGA5 and ITGB1, but not affected by siRNAs targeting mutp53 and ENTPD5 (Supplemental Fig. S3c-d).

The reduction in FN-adhesion translated into significantly reduced migration through FN-coated porous membranes and diminished invasion through FN-supplemented collagen gels in transwell (Boyden chamber) assays (Fig. 2b). Although FN-independent migration and invasion were also affected, the effect was significantly weaker (Fig. 2b). Importantly, live cell imaging revealed no proliferation defects in tumor cells depleted of either mutp53 or ENTPD5, excluding the possibility that the results from migration and invasion assays were confounded by proliferation effects (Supplemental Fig. S3e-f).

To confirm the proposed mutp53-ENTPD5 hierarchy, we conducted experiments to test the effects of enforced expression of ENTPD5 on adhesion, migration, and invasion in mutp53- or ITGA5-depleted cells. We found that enforced expression of ENTPD5 was sufficient to fully restore FN-mediated adhesion of mutp53-depleted but not of ITGA5-depleted cells (Fig. 2c). Similarly, enforced ENTPD5 expression also rescued FN-dependent migration and invasion of mutp53-depleted cells, suggesting that ENTPD5-mediated upregulation of ITGA5 protein levels is necessary and sufficient to mediate the pro-migratory and pro-invasive function of mutp53 (Fig. 2d-e). Interestingly, the smaller effect of mutp53 depletion on FN-independent migration and invasion was also fully rescued by ectopic ENTPD5 expression (Fig. 2d), indicating that the mutp53-ENTPD5 axis also controls migratory and invasive behavior in the absence of FN, albeit to a lesser extent. We conclude that the mutp53-ENTPD5 axis controls the binding of tumor cells to fibronectin and promotes their migration and invasion primarily through the upregulation of ITGA5 expression, without affecting cell proliferation.

### CANX/CALR cycle activity is essential for mutp53-driven ITGA5 function

**Fig. 3:**
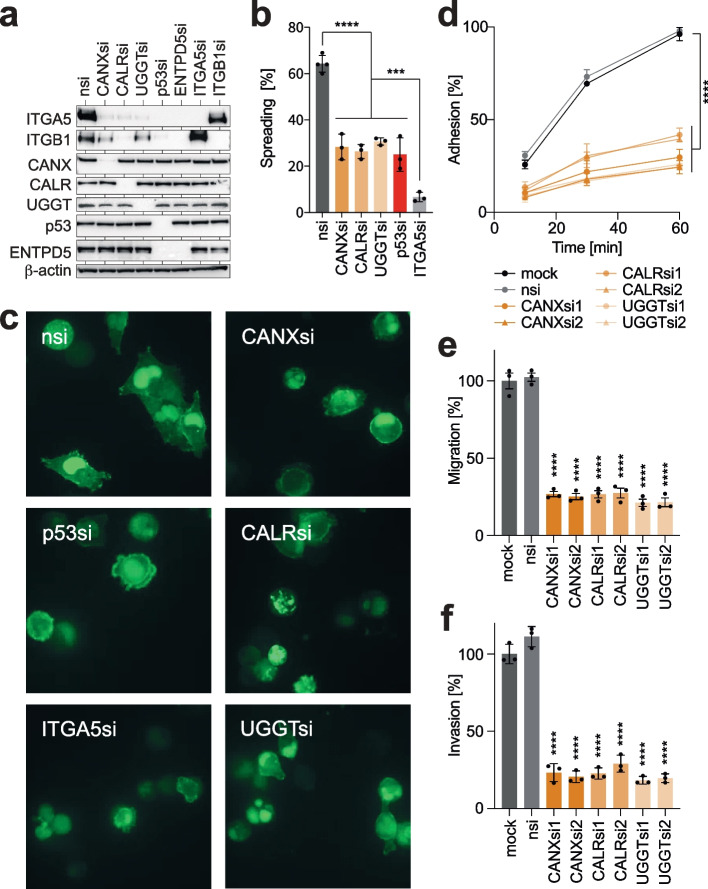
CANX/CALR chaperones are essential for mutant p53-dependent ITGA5 activity. **a** Western blot of MIA PaCa-2 cells following siRNA-mediated depletion of CANX, CALR, UGGT, p53, ITGA5, or ITGB1. **b**-**c** Cell-spreading assay. MIA PaCa-2 cells were depleted of indicated proteins and analyzed by spinning disc microscopy 45 min after plating on FN-coated slides: **b** percentage of spread cells shown as mean ± SD (*n* = 3 transfections with 125–200 counted cells per transfection); **c** representative images. **d-f** FN-mediated adhesion, migration, and invasion of MIA PaCa-2 cells depleted of CANX, CALR, or UGGT. The analysis was performed as described in Fig. 2. Results are shown as mean ± SD (*n* = 3 independent experiments). Statistical significance was tested using two-way (**d**) or one-way (**e–f**) ANOVA followed by Dunnett’s multiple comparisons test: *, *p* < 0.05; **, *p* < 0.01; ***, *p* < 0.001; ****, *p* < 0.0001; ns, not significant. Mock: non-transfected cells; nsi: non-targeting control siRNA

Folding of nascent N-glycoproteins, such as ITGA5 and ITGB1, occurs in the ER by the CANX/CALR chaperones [29]. Unfolded N-glycoproteins are recognized and tagged with a single glucose moiety by UGGT, which uses UDP-glucose as a substrate. The mutp53-driven ENTPD5 activity generates UMP, which is supposed to assist the CANX/CALR chaperone cycle by enabling the ER import of UDP-glucose in exchange for UMP [28]. Using confocal immunofluorescence microscopy, we confirmed the localization of ENTPD5 in the ER by co-localization with the ER marker protein disulfide isomerase family A member 3 (PDIA3) (Supplemental Fig. 4). ITGB1 is an obligate substrate of PDIA3 (better known as ERp57) in association with CANX/CALR and cannot fold efficiently in its absence or when ERp57 is present but the interaction with CANX/CALR is prevented [48]. To directly assess the role of the CANX/CALR chaperones and UGGT for high-level expression of ITGA5 and ITGB1 in p53-mutant cancer cells, we depleted CANX, CALR, and UGGT using RNAi. Depletion of any of these components reduced the expression of the respective targets and simultaneously diminished ITGA5 and ITGB1 protein expression (Fig. 3a). The results indicate that the mutp53-driven high-level expression of ITGA5/B1 in MIA PaCa-2 cells requires not only ENTPD5 but also CANX, CALR, and UGGT.

To investigate the functional role of the CANX/CALR cycle in ITGA5/B1-mediated adhesion to FN, we used MIA PaCa-2 cells expressing an actin-chromobody tagged with GFP to analyze the spreading of tumor cells on FN-coated plates by live-cell imaging. Control cells attached quickly and showed extensive spreading on FN within 45 min, while depletion of mutp53, ENTPD5, CANX/CALR, UGGT, or ITGA5 significantly decreased the percentage of spread cells (Fig. 3b), resulting in a rounder morphology with a smaller cell diameter (Fig. 3c).

Similarly, the depletion of single components of the ER quality control cycle also resulted in reduced adhesive, migratory, and invasive capacity of the cells in the presence of FN (Fig. 3d-f and Supplemental Fig. S5a) and to a lesser extent also in its absence (Supplemental Fig. S5b). Enforced expression of ENTPD5 failed to rescue FN adhesion in cells depleted of UGGT, CANX, or CALR, confirming that ENTPD5 acts upstream of the CANX/CALR cycle (Supplemental Fig. S5a). In summary, our results demonstrate that proper functioning of the ER quality control cycle is crucial for ITGA5 functions in adhesion, migration, and invasion and that the mutp53-ENTPD5 axis promotes ITGA5 functions via the CANX/CALR cycle.

### ITGA5-blocking antibodies inhibit mutp53-driven pro-metastatic properties

**Fig. 4:**
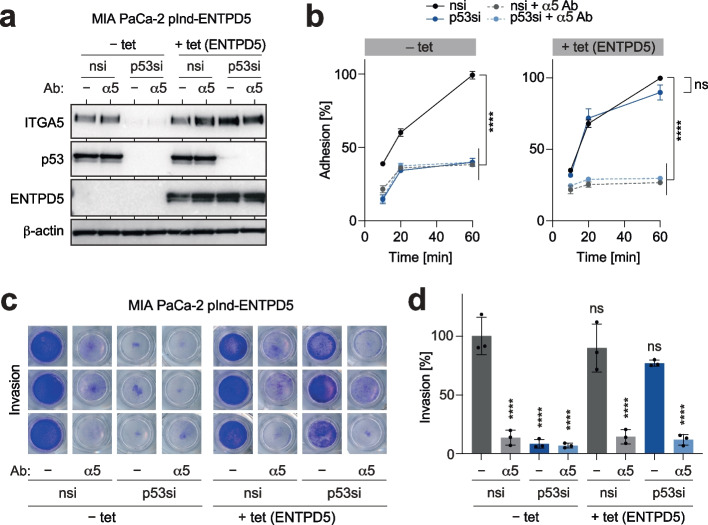
ITGA5-antibodies block mutant p53-driven pro-metastatic properties. **a** Western blot for p53, ENTPD5, and ITGA5. MIA PaCa-2 pIND-ENTPD5 cells (with tet-inducible expression of ENTPD5) were induced with doxycycline (+ tet), transfected with p53 siRNA (p53si) and treated with ITGA5-blocking antibody (α5) as indicated. β-actin is shown as a loading control. **b** Adhesion kinetics of MIA PaCa-2 pIND-ENTPD5 cells on FN. Cells were induced with doxycycline (+ tet) as indicated, transfected with p53 siRNA (p53si), and treated with ITGA5-blocking antibody (α5). **c-d** Invasion of MIA PaCa-2 pIND-ENTPD5 cells through FN-containing collagen gels following depletion of p53 and/or treatment with ITGA5-blocking antibody in the absence or presence of tet (ENTPD5 overexpression). **c** Representative images of crystal violet-stained transwell inserts. **d** Quantification of invasion assays. All graphs show the mean ± SD of *n* = 3 biological replicates. Statistical significance was tested using two-way ANOVA followed by Dunnett’s multiple comparisons test: ****, *p* < 0.0001; ns, not significant

As integrins are cell surface proteins, they can be inhibited with antibodies. We, therefore, investigated the potential of ITGA5-antibodies (Ab) as therapeutics to block mutp53-mediated pro-metastatic activities. Treating cells with an ITGA5-blocking Ab did not affect the expression of mutp53 and ENTPD5, which is consistent with ITGA5 being downstream of the mutp53-ENTPD5 axis (Fig. 4a). Nevertheless, ITGA5 blockade significantly decreased FN-mediated adhesion and invasion to a similar extent as direct depletion of mutp53 (Fig. 4b-d). Moreover, the addition of ITGA5-blocking Ab to mutp53-depleted cells did not further reduce adhesion and invasion, highlighting the dominant role of mutp53 as a driver of ITGA5-mediated pro-metastatic activity (Fig. 4b-d). Interestingly, enforced expression of ENTPD5 only rescued adhesion and invasion of mutp53-depleted cells in the absence of ITGA5-blocking Ab (Fig. 4b-d). These findings confirm the proposed hierarchy, underscore the exquisite dependence of ENTPD5's pro-metastatic functions on ITGA5, and validate ITGA5 as a potential therapeutic target downstream of the mutp53-ENTPD5 axis.

### α-glucosidase inhibitors block mutp53-dependent ITGA5 expression and function

**Fig. 5:**
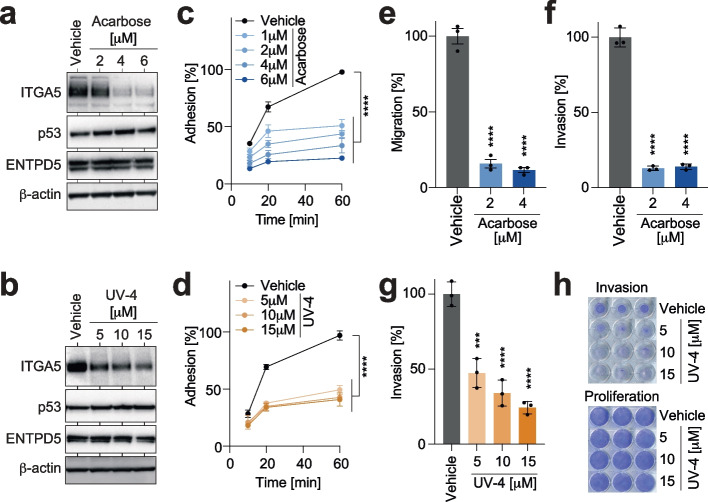
α-glucosidase inhibitors block mutant p53-dependent ITGA5 expression and function. **a-b** Western blot for p53, ENTPD5, and ITGA5 of MIA PaCa-2 cells treated with the indicated doses of the α-glucosidase inhibitors Acarbose and UV-4. **c-d** Adhesion kinetics on FN of Acarbose- and UV-4-treated MIA PaCa-2 cells. **e–g** FN-dependent transwell migration and invasion of MIA PaCa-2 cells treated with Acarbose and UV-4 as indicated. **h** Representative images of crystal violet-stained transwell inserts (invasion) and proliferation controls, documenting comparable cell seeding and viability. All graphs show the mean ± SD of *n* = 3 biological replicates. Statistical significance was tested using two-way ANOVA followed by Dunnett’s multiple comparisons test: ***, *p* < 0.001; ****, *p* < 0.0001; ns, not significant

To investigate the possibility of targeting the N-glycoprotein folding machinery as a therapeutic strategy to counteract the pro-metastatic effects of mutp53, we examined inhibitors of the α-glucosidases I and II. These enzymes sequentially trim the immature N-linked oligosaccharide core to a monoglucosylated glycan that is capable of binding to the chaperones CANX and CALR [29, 31, 49]. We hypothesized that α-glucosidase inhibitors could reduce the binding of unfolded ITGA5 precursors to the chaperones and, in turn, mitigate the consequences of increased CANX/CALR cycle activity. To test our hypothesis, we evaluated two chemically distinct classes of inhibitors: acarbose, a widely used antidiabetic drug that inhibits intestinal α-glucosidases [50], and UV-4, an antiviral iminosugar that inhibits endoplasmic α-glucosidases required for the maturation of viral glycoproteins [51–53]. Treating the tumor cells with either of the drugs resulted in a dose-dependent reduction of ITGA5 protein expression, but not mRNA expression (Fig. 5a-b and Supplemental Fig. S6a). Furthermore, treated tumor cells showed significantly reduced FN-mediated, and to a lesser degree, FN-independent, adhesion, migration, and invasion (Fig. 5c-h and Supplemental Fig. S6b-d), while cellular proliferation was not affected (Supplemental Fig. S6e-f). Expectedly, the ITGA5 dysfunction, due to inhibited entry into the chaperone cycle was not rescued by enforced expression of ENTPD5 (Supplemental Fig. S6b). These findings further underline the importance of the N-glycoprotein folding machinery for pro-metastatic properties of mutp53 and highlight the potential of available α-glucosidase inhibitors as a potential therapeutic strategy for p53-mutant tumors.

### Pharmacological degradation of mutp53 inhibits ITGA5-mediated cancer cell motility in vitro

**Fig. 6:**
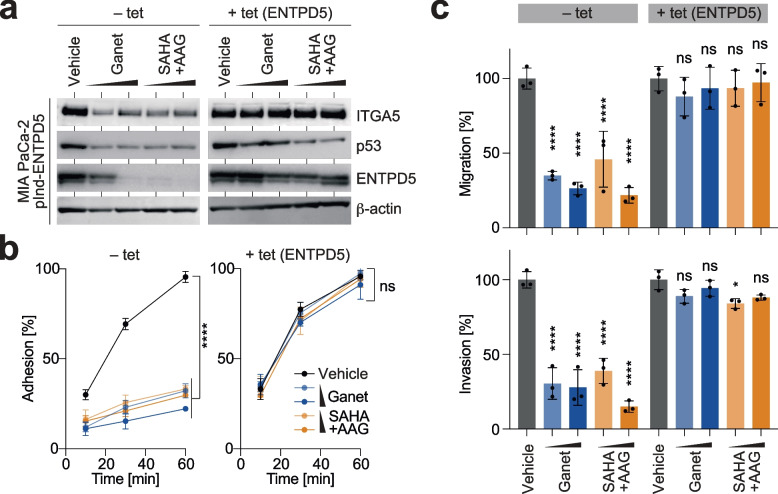
Degradation of mutant p53 blocks ITGA5-mediated cancer cell motility in vitro*.* MIA PaCa-2 pIND-ENTPD5 cells (with tet-inducible expression of ENTPD5) were induced with doxycycline (+ tet) as indicated and treated with Ganetespib (Ganet, 0.5–1.0 µM), SAHA/17-AAG (10–15 µM) or vehicle. **a** Western blot for p53, ENTPD5 and ITGA5. β-actin is shown as a loading control. **b** Adhesion kinetics on FN. **c** FN-mediated transwell migration and invasion. All graphs show the mean ± SD of *n* = 3 biological replicates. Statistical significance was tested using two-way ANOVA followed by Dunnett’s multiple comparisons test: ****, *p* < 0.0001; ns, not significant

To investigate the effect of drugs targeting the mechanisms responsible for the aberrant stabilization of mutant p53 in cancer cells, specifically HSP90 and its indispensable regulator HDAC6 [11, 12, 54], we examined two HSP90 inhibitors, 17-N-allylamino-17-demethoxygeldanamycin (AAG) and Ganetespib (Ganet), as well as the potent HDAC inhibitor Suberoylanilide hydroxamic acid (SAHA). Treating MIA PaCa-2 cells with either Ganet or the combination of SAHA/AAG effectively reduced the mutp53 protein level along with a marked decrease in ENTPD5 and ITGA5 protein, but not *ITGA5* mRNA levels (Fig. 6a, left panel, and Supplemental Fig. S7a), confirming that high-level ITGA5 expression is dependent on the constitutive stabilization of mutp53 by heat shock chaperones. Moreover, there was no impact of Ganet on ENTPD5 and ITGA5 in p53-null H1299 cells (Supplemental Fig. S7b), indicating that both treatments act on ITGA5 in a mutp53-specific manner. Enforced expression of ENTPD5 did not diminish the drug-induced degradation of mutp53 but effectively prevented the downregulation of ITGA5 (Fig. 6a, right panel). This underscores the critical role of ENTPD5 downregulation in causing ITGA5 loss in response to the mutp53-destabilizing drug treatment.

The treatment effects on ITGA5 protein levels had no noticeable effect on cell proliferation (Supplemental Fig. S7c-d). However, it resulted in a direct and dose-dependent decrease in adhesion, migration, and invasion (Fig. 6b-c, left panels). This effect was rescued by re-expression of ENTPD5 (Fig. 6b-c, right panels) and was not observed in p53-null cells (Supplemental Fig. S7e). Notably, the inhibitory effect of Ganet on adhesion and invasion was comparable to that of ITGA5-blocking antibodies, and there was no additive effect when both treatments were combined (Supplemental Fig. S7f-h). These findings are consistent with Ganet and ITGA5-Ab acting on the same pathway with similar efficacy. The rescue effect of ENTPD5 was abolished by ITGA5-Ab, further validating the hierarchical regulation of ITGA5 by mutp53 through ENTPD5 (Supplemental Fig. S7f-h).

### Ganetespib inhibits PDAC ITGA5 expression and metastasis in an ENTPD5-dependent manner

**Fig. 7:**
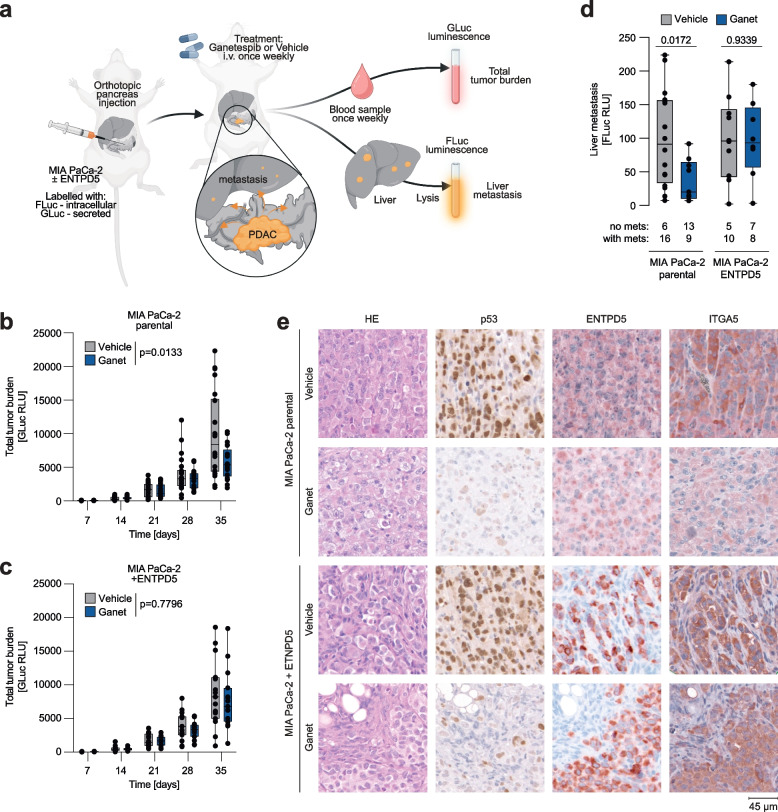
Targeting mutp53-ENTPD5 signaling inhibits ITGA5 expression, tumor growth, and metastasis in a preclinical PDAC model. **a** PDAC model: parental MIA PaCa-2 and MIA PaCa-2 pIND-ENTPD5 cells (with tet-inducible expression of ENTPD5) were ex vivo labeled with a tandem GLuc-FLuc construct and orthotopically injected into the pancreas of immunodeficient mice. Mice were treated with either Ganetespib (Ganet) or vehicle. All mice received doxycycline. Total tumor growth was measured by blood levels of GLuc. Liver metastasis was quantified by FLuc measurement of liver lysates. Image created with BioRender.com. **b-c** Total tumor growth in mice transplanted with (**b**) parental (*n* = 22 each group) and (**c**) ENTPD5-overexpressing MIA PaCa-2 tumors (*n* = 15 each group). Shown is GLuc blood activity in relative light units (RLU) as a surrogate marker of the total tumor burden. **d** Liver metastasis is shown as FLuc activity (RLU, relative light units) of liver lysates. The number of animals with and without liver metastases in each group is indicated below the graph. **e** Pancreatic mouse tumors were analyzed by HE staining and immunohistochemistry for (mutant) p53, ENTPD5, and ITGA5. All boxplots show the minimum value, the first quartile, the median, the third quartile, and the maximum value. Single data points represent individual mice. Statistical significance was tested using two-way ANOVA followed by Dunnett’s multiple comparisons test

To investigate the role of mutp53 for ITGA5 expression and metastasis in vivo, we used an orthotopic xenograft model based on MIA PaCa-2 cells with or without tet-inducible ENTPD5 expression. For monitoring, the cells were transduced in vitro with the secreted *Gaussia princeps* luciferase (GLuc) and the intracellular firefly luciferase (FLuc). After dual labeling, both cell types were surgically transplanted into the pancreata of immunodeficient mice (Fig. 7a). All mice received doxycycline continuously via the drinking water, maintaining stable ENTPD5 expression levels in mice transplanted with MIA PaCa-2 cells with tet-inducible ENTPD5. Mice were divided into two treatment groups, receiving weekly doses of Ganet or solvent as a control. Tumor burden was measured weekly based on the increase in GLuc blood levels (Fig. 7b-c). Four weeks post-transplantation, primary tumors were explanted and histologically analyzed. Livers, as the primary metastatic organs, were collected, lysed, and assessed for firefly luciferase activity (Fig. 7d). Immunohistochemical analysis revealed efficient mutp53 degradation in both Ganet-treated mouse cohorts and increased ENTPD5 expression in tumors derived from MIA PaCa-2 cells with tet-inducible ENTPD5 (Fig. 7e). Ganet treatment significantly downregulated ITGA5 levels in parental MIA PaCa-2 tumors (Fig. 7e). Confirming in vivo that ITGA5 expression levels are controlled by the mutp53-ENTPD5 axis, downregulation of ITGA5 expression by Ganet was rescued by tet-induced ENTPD5 expression (Fig. 7e). Concurrently, Ganet treatment substantially reduced both total tumor burden (*p* = 0.0133, Fig. 7b-c) and liver metastasis (*p* = 0.018, Fig. 7d) without significant effects on tumors with stable, mutp53-independent ENTPD5 expression (Fig. 7b-d). Collectively, these results validate in an orthotopic in vivo model that mutp53 promotes metastasis via ENTPD5 and underscore the efficacy of Ganet in targeting pro-metastatic mutp53 effector functions that are mediated by ENTPD5 and ITGA5.

## Discussion

Over half of all cancer patients harbor p53 missense mutations, many of which switch the protein from a tumor suppressor to a tumor-promoting oncogene empowered with GOF activities. These activities are associated with increased metastasis, chemoresistance, and decreased survival in both mice and patients [4–6, 55]. While this highlights mutant p53 as an attractive therapeutic target and has spurred research into targeting strategies, establishing the feasibility of mutp53-targeting in clinical settings is still a challenge [11–13, 56]. Therefore, targeting potentially better drug-responsive effectors of mutp53 remains a promising alternative approach for cancer therapy.

Our previous research has described ENTPD5 as a key effector of the pro-invasive and pro-metastatic mutp53 GOF [25]. ENTPD5 is an ER-resident UDPase that promotes the proper folding of N-glycoproteins through the CANX/CALR cycle [28]. This glycoprotein folding cycle is an essential quality control mechanism, ensuring that only correctly folded and functional glycoproteins exit the ER, are further processed by the Golgi, and transported to the cell membrane [29, 57]. Among the heavily glycosylated glycoproteins, receptor tyrosine kinases play crucial roles in various aspects of tumorigenesis [34]. Another group of heavily N-glycosylated cell membrane proteins are integrins; a family of heterodimeric transmembrane receptors that promote bidirectional communication of cells with their microenvironment [47, 58].

In this study, we have delineated a novel pro-metastatic pathway in cancer cells that is stimulated by mutp53. We found that ENTPD5, a downstream target of mutp53 [25], plays a crucial role in enhancing the expression of the integrins ITGA5 and ITGB1 (Fig. 1 and Supplemental Fig. 1). Focusing on ITGA5, we demonstrated that its increased expression relies on the presence of UGGT as well as the chaperones CANX and CALR (Fig. 3a). Importantly, the elimination of ENTPD5 from the cells, either directly or indirectly, by depleting or destabilizing mutp53, resulted in a significant reduction in ITGA5 protein expression. However, the reintroduction of ENTPD5 expression, even in the absence of mutp53, restored ITGA5 expression. We observed the regulation of ITGA5 by the mutp53/ENTPD5 axis across various clinically relevant cancer types, including pancreatic, lung, and breast cancer and for multiple different p53 missense mutants (Supplemental Fig. 1). Interestingly, in ovarian cancer cells, similar levels of ITGA5 and ITGB1 were expressed, but this expression was not dependent on mutp53 or ENTPD5. In these ovarian cancer cells, FN-adhesion was dependent on ITGA5/B1, but independent of ENTPD5, suggesting potential tissue- or cell type-specific requirements for ENTPD5 in integrin folding. Considering the clinical impact of ITGA5 and ITGB1 expression on the survival of PDAC patients (Supplemental Fig. 2), we focused our subsequent experiments on this cancer type, for which better treatment options are urgently required due to its poor survival outlook [59].

Integrins serve as primary cell adhesion receptors for components of the extracellular matrix (ECM). They dictate the specific ECM components a cell can adhere to, thereby influencing how a cell senses and reacts to external signals. Cancer cells may lose their original tissue attachment and inevitably undergo changes in cell-to-cell and cell-to-ECM adhesion. These alterations can lead to the entry of cancer cells into the bloodstream or lymphatic system, extravasation, and ultimately the formation of metastatic colonies in distant locations [60, 61]. In such scenarios, cancer cells not only exploit the standard integrin-mediated adhesion functions for migration but also manipulate their surrounding microenvironment to their advantage. Consequently, alterations in various integrin-signaling pathways or changes in integrin expression patterns have been associated with numerous types of cancer [60, 61].

ITGA5 pairs with ITGB1 to form the heterodimeric integrin α5β1, the primary receptor for FN, a critical component in the formation of the ECM meshwork [62]. The high affinity and specificity of α5β1 for the RGD motif of FN is mediated by a specific residue (Asp154) in the extracellular ITGA5 domain [63]. In addition, integrins undergo conformational changes from a bent to an extended conformation that increase their affinity [64]. Increasing evidence also suggests a connection between lipid raft microdomains and integrin activation in the context of mechanotransduction [65]. When integrin-α5β1 binds to FN, this triggers bidirectional signaling which contributes to cancer progression by driving survival, proliferation, migration, and invasion [66, 67].

Given the central role of the CANX/CALR cycle in glycoprotein biosynthesis, it can be speculated that the stimulating effect of mutp53 on the ER chaperones extends beyond ITGA5 and ITGB1. Other integrins may also experience a similar boost in their expression. However, due to the inverse correlation of ITGA5 and ITGB1 with the survival of PDAC patients (Supplemental Fig. 2), we focused on investigating FN-mediated cell motility and its regulation through the mutp53-ENTPD5-ITGA5 axis. Using FN as the extracellular matrix, our adhesion, migration and invasion experiments demonstrated that cells depleted of ENTPD5 or mutp53 lose their ability to interact with FN (Fig. 2). These effects were comparable to cells directly deprived of ITGA5 (Fig. 2) or treated with ITGA5-blocking antibodies (Fig. 4). These effects were all dependent on the CANX/CALR chaperones (Fig. 3). Additionally, in ovarian cancer cells, where mutp53 or ENTPD5 depletion did not affect ITGA5 and ITGB1 expression levels (Supplemental Fig. 1), we observed no inhibition of FN binding by mutp53 or ENTPD5 depletion (Supplemental Fig. 3). This not only strengthens the view that the mutp53-ENTPD5 axis functionally regulates ITGA5 and ITGB1 but also provides new insights into how mutp53 profoundly modulates tumor-stroma interactions to create a pro-metastatic environment by regulating the ER quality control machinery.

Interestingly, p53 mutations have been found to impact glycoprotein biosynthesis at multiple levels, highlighting the pivotal role of glycoproteins as pro-metastatic effectors [9]. Certain receptor tyrosine kinases, such as EGFR or PDGFRα, are directly induced at the transcriptional level by mutp53 through interactions with transcription factors like Sp1, NF-Y, or p73 [22, 68]. Additionally, mutp53 interacts with its family member p63 to transcriptionally induce the Rab-coupling protein (RCP), which facilitates the endosomal recycling of EGFR, MET, and integrins [23, 69, 70]. Moreover, mutp53-expressing tumor cells modulate exosome secretion, influencing integrin recycling in neighboring tumor cells and normal fibroblasts, thereby promoting the deposition of a highly pro-invasive extracellular matrix at both the primary tumor site and pre-metastatic niches in distant organs [71]. By interacting with the hypoxia-responsive factor HIF1α, mutp53 induces tubulo-vesiculation of the Golgi apparatus, leading to enhanced vesicular trafficking, secretion of soluble factors, and deposition and remodeling of the ECM [24]. Notably, modulation of secretory vesicle biogenesis in the Golgi has also been reported as a consequence of p53 inactivation, even in the absence of mutp53-dependent GOF properties [72, 73]. As such, the transcriptional upregulation of cell surface proteins by mutp53 exhibits mechanistic diversity and poses challenges for targeted interventions. However, the chaperone-mediated folding of nascent glycoproteins in the ER represents a central and universal step in the production of most cell membrane and secreted proteins. This highlights the potential of targeting the mutp53-ENTPD5-mediated control of the CANX/CALR cycle as an attractive therapeutic strategy.

In this study, we have explored different approaches to target the mutp53-ENTPD5-ITGA5 axis. We employed strategies such as ITGA5-blocking antibodies, inhibition of the N-glycoprotein folding chaperone machinery in the CANX/CALR cycle, and mutp53 degradation. Our experiments utilizing specific blocking antibodies against ITGA5 confirmed its crucial role as a downstream effector of mutp53-driven motility (Fig. 4). However, clinical testing of integrin-targeting antibodies has yielded rather disappointing results [74–76]. Several factors contribute to the therapeutic failure, including variable expression of integrins, functional redundancy among integrins, distinct roles of integrins at different disease stages, and the sequestration of therapeutics by integrin-containing tumor-derived extracellular vesicles [76]. In fact, we also observed mutp53-induced expression of ITGA5 on extracellular vesicles in our model, which may impact the efficiency of blocking antibodies.

Therefore, we decided to investigate targeting the ER chaperone machinery as our next approach. As direct inhibitors of CANX or CALR were not available, we focused on inhibiting α-glucosidases I and II, which sequentially trim the core oligosaccharide on nascent N-glycoproteins to generate the monoglucosylated protein recognized by CANX and CALR [29]. Glucosidase II also removes the remaining single glucose moiety, facilitating the release of proteins from the chaperones [29]. By inhibiting ER-resident glucosidases, the entry into and exit from the CANX/CALR cycle are blocked. Consequently, the proteins are unable to attain their native conformation and do not pass ER quality control [77]. These misfolded proteins are retained in the ER and ultimately undergo degradation [78, 79]. It may be expected that glucosidase inhibitors have significant side effects in normal cells since their function is crucial for proper protein folding. However, not all glycoproteins are equally dependent on glycans for folding and secretion [29]. In the absence of glycans, many glycoproteins experience only a partial loss of folding and secretion efficiency, while others become temperature-sensitive or remain unaffected [29]. As a rule, glycoproteins with a larger number of glycans rely more heavily on the chaperone machinery, suggesting a potential therapeutic window that has also been observed when targeting glycoprotein processing for antiviral therapy. Many viruses require the host ER protein-folding machinery in order to correctly fold one or more of their glycoproteins [80]. Iminosugars, such as UV-4, inhibit the ER-resident α-glucosidases I and II, interfere with proper folding of viral proteins, and display broad-spectrum activity against multiple viruses including SARS-CoV-2 [51, 53, 80–84]. Early clinical trials have demonstrated the good tolerability of iminosugars in patients, with minor and reversible osmotic diarrhea being the main side effect due to the inhibition of α-glucosidases in the gut [81]. It is worth noting that the widely used antidiabetic drug Acarbose acts through inhibition of intestinal α-glucosidases [85]. In our study, both the iminosugar UV-4 and Acarbose showed in vitro efficacy in reducing ITGA5 expression and FN-mediated motility in PDAC cells without negatively affecting tumor cell viability and proliferation (Fig. 5 and Supplemental Fig. 6). However, Acarbose, despite being a clinically approved drug and effective α-glucosidase inhibitor in cell culture, acts locally in the gastrointestinal tract. It has low systemic bioavailability, making it unsuitable for systemic cancer therapy [86]. On the other hand, iminosugars exhibit a promising therapeutic window as antiviral agents and show a high tolerability in animal infection models. Therefore, they should be further investigated for anti-metastatic activity in preclinical cancer models.

To directly target the mutp53-ENTPD5-integrin axis at the level of mutp53, we investigated compounds that target the heat shock chaperone machinery, specifically HSP90 and its indispensable regulator HDAC6. These chaperones play a crucial role in stabilizing mutp53, which is considered essential for the GOF activity of mutp53 in cancer cells [15–19]. Building on previous research, we examined the HSP90 inhibitors, AAG and Ganet, as well as the potent HDAC inhibitor SAHA [15–19]. Treating cancer cells harboring p53 missense mutations, but not null mutations, with any of these drugs resulted in the destabilization of mutp53 and the simultaneous loss of ENTPD5 and ITGA5 expression (Fig. 6). This finding indicates that the elevated levels of ITGA5 are dependent on the aberrant stabilization of mutp53. Notably, these drugs strongly impaired the cells’ ability to interact with FN for adhesion, migration or invasion. The drug effects were largely restored by enforced expression of ENTPD5, validating that the diminished tumor cell motility was a result of compromised ENTPD5 activity. Importantly, we confirmed these observations in a preclinical model, by treating mice with orthotopic PDAC xenografts with Ganet. The treatment not only reduced mutp53 levels but also concurrently diminished ENTPD5 and ITGA5 expression. Emphasizing the crucial role of ENTPD5, the ectopic expression of ENTPD5 rescued the effects of the drug (Fig. 7). Although we did not observe a negative effect of Ganet on cell proliferation in 2D cell culture (Supplemental Fig. 7c-d), treatment with the drug led to a reduction in both primary tumor growth and metastatic spread (Fig. 7b-d). It is worth noting that mutant p53 (or ENTPD5) depletion by RNAi has been shown to decrease 2D colony formation in MIA PaCa-2 cells [25, 87], suggesting that mutp53 specifically promotes proliferation under clonogenic growth conditions. Interestingly, mutp53 was found to stimulate FN-independent migration of MIA PaCa-2 and other R248W-mutant PDAC cells, possibly through the formation of a complex between mutp53 and phosphorylated STAT3 [88]. This FN-independent migration was blocked by HSP90 inhibition as well [88]. In our study, we also observed some degree of stimulation of FN-independent motility by the mutp53-ENTPD5-CANX/CALR axis. However, the effects were much less pronounced compared to those mediated by ITGA5 or FN (Fig. 2b and d, and Supplemental Fig. 5b). However, considering the central role of ER chaperones in glycoprotein biosynthesis, we cannot exclude an additional role of the mutp53-ENTPD5 axis for expression of JAK receptors and JAK/STAT signaling, which potentially synergizes with the reported downstream effects of mutp53 on STAT3 activity [19, 88].

Although we observed the mutp53-ENTPD5-dependent ITGA5 expression in different cancer cell lines, our primary focus was on PDAC due to the clear prognostic implications of ITGA5 and its partner ITGB1 in this particular cancer type. However, we previously identified the role of the mutp53-ENTPD5 axis in promoting lung metastasis of breast cancer and now observed the regulation of ITGA5 and ITGB1 by this axis in the same model (Supplemental Fig. 1a and i). Hence, it is tempting to speculate that this pathway may also promote breast cancer metastasis to the lung through its impact on ITGA5 expression.

As our study was conducted using cell cultures and preclinical mouse models, further research is needed to determine the translatability of these findings to clinical settings. Although HSP90 inhibitors and glucosidase-blocking iminosugars have shown promising results in terms of safety in preclinical and early-stage clinical studies [80, 89–91], their efficacy in cancer therapy still needs to be established through further preclinical and clinical trials. The GANNET53 trial, a small phase I/II study for platinum-resistant ovarian cancer, combined paclitaxel with Ganetespib to target mutant p53 and showed no dose-limiting toxicities, leading to its selection for a randomized phase II trial. Novel HSP90 inhibitors are continuously being discovered and tested for their antitumor efficacy in preclinical and clinical trials, raising hope for the development of even more potent drugs to target mutp53 stabilization.

## Conclusions

**Fig. 8:**
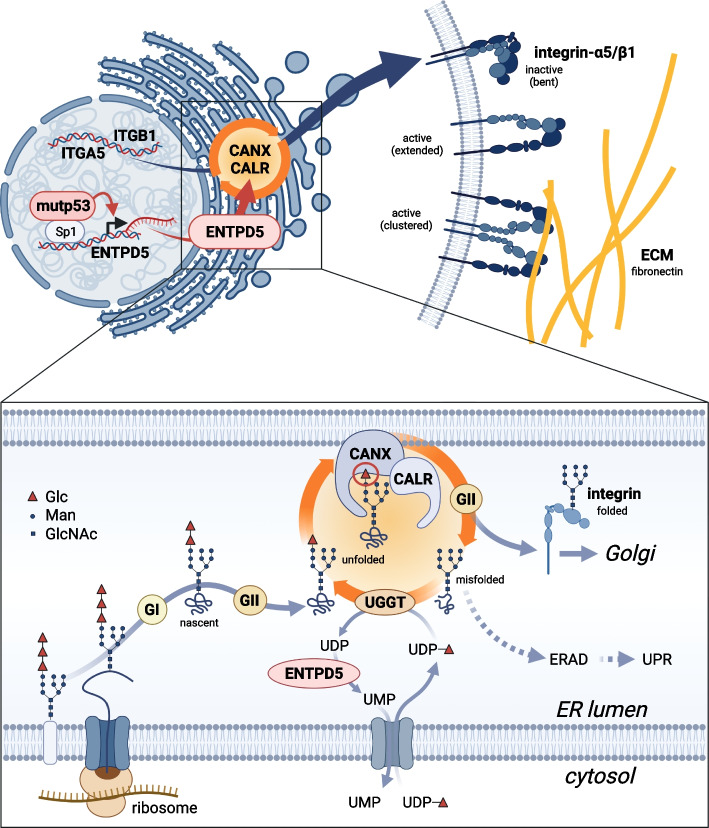
Graphical abstract illustrating the mechanism of mutp53-driven integrin-α5/β1 expression. Mutant p53 (mutp53) proteins induce the transcription of ectonucleoside triphosphate diphosphohydrolase 5 (ENTPD5). ENTPD5 operates in the endoplasmic reticulum as a UDPase to generate UMP. Subsequently, UMP is exchanged for UDP-glucose, which is required by UDP-glucose:glycoprotein glucosyltransferase (UGGT) to add a single glucose moiety (represented by a red triangle) to unfolded N-glycoproteins, including integrins. The presence of a single, terminal glucose residue enables the interaction with the lectin chaperones calnexin (CANX) and calreticulin (CALR), promoting proper folding. α-glucosidases I and II (GI, GII) trim glucose moieties and, along with UGGT, regulate the entry and exit of integrins from the CANX/CALR cycle. Once correctly folded, the integrins exit the ER and are transported to the cell membrane through the Golgi apparatus. To avoid the accumulation of misfolded proteins, cells employ the endoplasmic reticulum-associated degradation (ERAD) pathway or activate the unfolded protein response (UPR) mechanisms. At the cell membrane, integrin-α5/β1 dimers are activated, adopting an extended conformation, and form clusters to mediate adhesion to fibronectin, a component of the extracellular matrix (ECM). This process promotes cellular migration, invasion, and metastasis. Created with BioRender.com

Our study has identified the heterodimeric ITGA5/B1 fibronectin receptor as a crucial downstream effector of the pro-metastatic activity of mutant p53 proteins. We found that ITGA5/B1 protein levels are increased by mutp53-driven expression of the ER-resident UDPase ENTPD5, which is crucial for the chaperone function of calnexin and calreticulin in N-glycoprotein folding (Fig. 8). The mutp53-ENTPD5-driven chaperone activity boosts FN-mediated tumor cell motility (adhesion, migration and invasion) in an ITGA5-dependent manner. Our results also show that high expression of the fibronectin receptor subunits ITGA5 and ITGB1 correlates with poor disease-free survival in patients with pancreatic adenocarcinoma. Targeting the mutp53-ENTPD5-ITGA5 axis with ITGA5-blocking antibodies or inhibitors of ER-resident α-glucosidases interferes with FN-mediated tumor cell motility in vitro. Destabilization of mutp53 with HSP90-inhibitors also blocks FN-mediated motility in vitro and, in addition, inhibits tumor growth and metastasis in an orthotopic PDAC mouse model.

In summary, our findings shed light on the molecular mechanisms underlying mutp53-driven metastasis and suggest potential therapeutic strategies for p53-mutant pancreatic adenocarcinoma. Further investigations are needed to validate the efficacy and safety of these therapeutic approaches in preclinical and clinical settings and to explore the implications for other types of cancer.

## Supplementary Information

**Additional file 1: Supplemental Figure S1.** Mutp53 regulation of ENTPD5, ITGA5, and ITGB1 in different cancer cell lines. **a-e** Western blot for p53, ITGA5, and ENTPD5, in p53-mutated breast (MDA-MB-231), lung (PC-9 and H1975) and ovarian (OC58 and OC121) cancer cells, transfected with siRNAs targeting p53 or ENTPD5 as indicated. nsi, non-targeting siRNA. **g** RT-qPCR analysis of *ITGA5* mRNA expression in MIA PaCa-2 cells transfected with siRNAs targeting p53, ENTPD5, CANX, CALR, UGGT or ITGA5. Shown is the log2-fold mean normalized expression (MNE) ± SD (n=3 replicates). Statistical significance was tested using one-way ANOVA followed by Dunnett’s multiple comparisons test versus control: ****, *p*<0.0001; ns, not significant. **g-j** Western blot for p53, ITGB1, and ENTPD5, in p53-mutated pancreatic (MIA PaCa-2), breast (MDA-MB-231), lung (H1975) and ovarian (OC58) cancer cells, transfected with siRNAs as indicated. nsi, non-targeting siRNA.

**Additional file 2: Supplemental Figure S2.** Kaplan-Meier plots showing disease-free survival for patients with the indicated cancer types (PDAC, pancreatic ductal adenocarcinoma; LUAD, lung adenocarcinoma; BRCA, breast cancer; OVCA, ovarian carcinoma) stratified into high vs. low *ITGA5* or *ITGB1* mRNA expressing groups. Plots were generated and data statistically analyzed with GEPIA2 [46].

**Additional file 3: Supplemental Figure S3.** Mutant p53 signaling via ENTPD5 is required for FN-mediated cell adhesion, migration, and invasion. **a-d** Adhesion kinetics of (**a**) H1975, (**b**) MDA-MB-231, (**c**) OC58 and (**d**) OC121 cells to fibronectin (FN) following siRNA-mediated depletion of p53, ENTPD5, ITGA5 or ITGB1. Adhesion is expressed as the percentage of the seeded cell number. **e-f** Proliferation effect of mutant p53 and ENTPD5. MIA PaCa-2 cells were transfected with siRNAs targeting p53 and ENTPD5 and analyzed by real-time live cell imaging. Non-targeting siRNA (nsi) is shown as control. **e** Confluence curves. Shown is the mean confluence of n=3 replicates. Shading for nsi-transfected cells indicates the SD. **f** Proliferation was quantified as the area under the confluence curve and normalized to nsi control cells as 100%. Shown is the mean ± SD (n=3 replicates)

**Additional file 4: Supplemental Figure S4.** ENTPD5 co-localizes with PDIA3 in the endoplasmic reticulum. Shown are representative confocal immunofluorescence microscopy images of MIA PaCa-2 cells stained with antibodies against the endoplasmic reticulum marker PDIA3 (protein disulfide isomerase family A member 3, also known as ERp57) and ENTPD5. Shown is the Pearson correlation coefficient for co-localization calculated with the Coloc 2 plug-in in ImageJ.

**Additional file 5: Supplemental Figure S5.** CANX/CALR chaperones are essential for mutant p53-dependent ITGA5 activity. **a** FN-mediated adhesion of MIA PaCa-2 cells with tet-inducible expression of ENTPD5. Cells were treated with doxycycline 24 hours before transfection of indicated siRNAs and analyzed as in Fig. 2. **b** Migration and invasion of MIA PaCa-2 cells in the absence and presence of FN following depletion of CANX, CALR, and UGGT.
All results are shown as mean ± SD (n=3 independent experiments). Statistical significance was tested using two-way ANOVA followed by Dunnett’s multiple comparisons test: ***, *p*<0.001; ****, *p*<0.0001; ns, not significant. Mock: non-transfected cells; nsi: non-targeting siRNA control.

**Additional file 6: Supplemental Figure S6.** α-glucosidase inhibitors block mutant p53-dependent *ITGA5* expression and function. **a** RT-qPCR analysis of *ITGA5* mRNA expression in MIA PaCa-2 cells treated with Acarbose and UV-4 as indicated. Shown is the GAPDH-normalized expression relative to vehicle-treated control cells as 100% (n=3 replicates). **b** Adhesion kinetics on FN of Acarbose-treated MIA PaCa-2 cells with tet-inducible ENTPD5 expression. **c-d** Migration and invasion of Acarbose-treated MIA PaCa-2 cells in the absence and presence of FN. **e-f** MIA PaCa-2 cells were treated with Acarbose or UV-4 and analyzed by real-time live cell imaging. **e** Confluence curves. Shown is the mean confluence of n=3 replicates. Shading for vehicle-treated control cells indicates the SD. **b** Proliferation was quantified as the area under the confluence curve and normalized to vehicle-treated control cells as 100%.
All results are shown as mean ± SD (n=3 replicates).

**Additional file 7: Supplemental Figure S7.** Degradation of mutant p53 blocks ITGA5-mediated cancer cell motility. **a** RT-qPCR analysis of *ITGA5* mRNA expression in MIA PaCa-2 cells treated with Ganet (0.5 or 1 μM) or SAHA+AAG (10 or 15 μM). Shown is the GAPDH-normalized expression relative to vehicle-treated control cells as 100% (n=3 replicates). **b** Western blot of p53-null H1299 cells for ITGA5 and ENTPD5 expression following treatment as in (**a**). **c-d** MIA PaCa-2 cells were treated as in (**a**) and analyzed by real-time live cell imaging. **c** Confluence curves. Shown is the mean confluence of n=3 replicates. Shading for vehicle-treated control cells indicates the SD. **d** Proliferation was quantified as the area under the confluence curve and normalized to vehicle-treated control cells as 100%. **e** Adhesion on FN of p53-null H1299 cells treated as in (**a**). **f-h** MIA PaCa-2 pIND-ENTPD5 cells (with tet-inducible expression of ENTPD5) were treated with doxycycline (+tet), Ganet (0.5 µM) and ITGA5-blocking antibody (α5) as indicated. **f** Adhesion on FN. **g-h** Invasion in the presence of FN. Statistical significance was tested using one-way ANOVA followed by Dunnett’s multiple comparisons test: ****, *p*<0.0001; ns, not significant.
All results are shown as mean ± SD (n=3 replicates).

## Data Availability

All data generated or analyzed during the present study are included in this published article.

## Abbreviations

PDAC: Pancreatic ductal adenocarcinoma
ITGA5: Integrin-α5
ITGB1: Integrin-β1
CANX: Calnexin
CALR: Calreticulin
mutp53: Mutant p53
ENTPD5: Ectonucleoside triphosphate diphosphohydrolase 5
LOF: Loss-of-function
GOF: Gain-of-function
Ganet: Ganetespib
SAHA: Suberoylanilide hydroxamic acid
17-AAG: 17-Allylamino-17-demethoxygeldanamycin
GLuc: *Gaussia princeps* Luciferase
FLuc: Firefly luciferase
