# Supplementary figures and images for: Mutant p53-ENTPD5 control of the calnexin/calreticulin cycle: a druggable target for inhibiting integrin-α5-driven metastasis
Source: J Exp Clin Cancer Res. 2023 Aug 10;42:203. doi: 10.1186/s13046-023-02785-z (PMC10413714; doi:10.1186/s13046-023-02785-z)

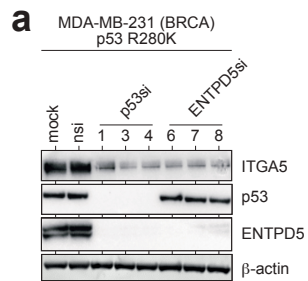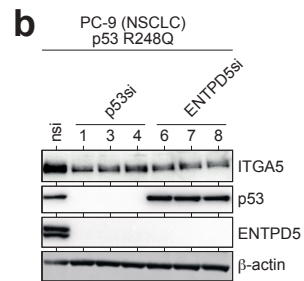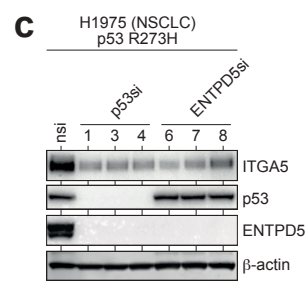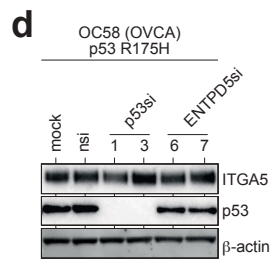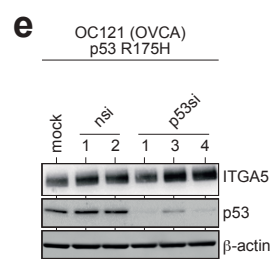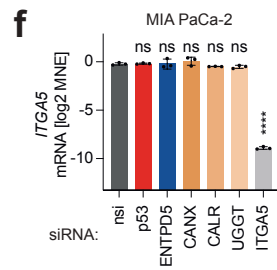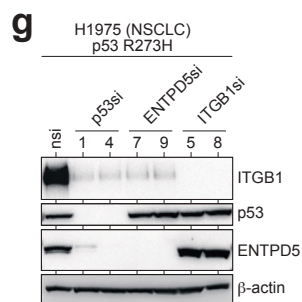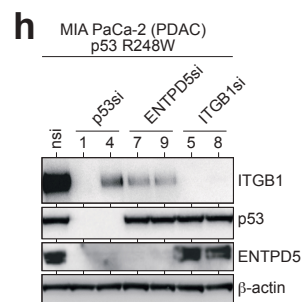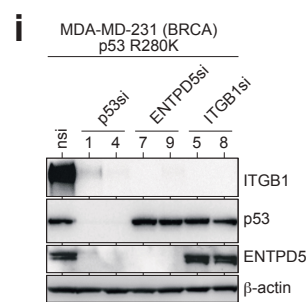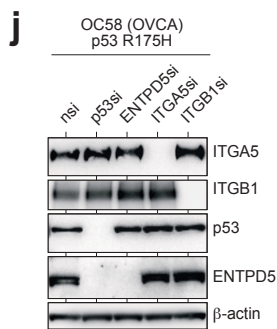

Supplement: Supplementary file 1 — Additional file 1: Supplemental Figure S1. Mutp53 regulation of ENTPD5, ITGA5, and ITGB1 in different cancer cell lines. a-e Western blot for p53, ITGA5, and ENTPD5, in p53-mutated breast (MDA-MB-231), lung (PC-9 and H1975) and ovarian (OC58 and OC121) cancer cells, transfected with siRNAs targeting p53 or ENTPD5 as indicated. nsi, non-targeting siRNA. g RT-qPCR analysis of ITGA5 mRNA expression in MIA PaCa-2 cells transfected with siRNAs targeting p53, ENTPD5, CANX, CALR, UGGT or ITGA5. Shown is the log2-fold mean normalized expression (MNE) ± SD (n=3 replicates). Statistical significance was tested using one-way ANOVA followed by Dunnett’s multiple comparisons test versus control: ****, p<0.0001; ns, not significant. g-j Western blot for p53, ITGB1, and ENTPD5, in p53-mutated pancreatic (MIA PaCa-2), breast (MDA-MB-231), lung (H1975) and ovarian (OC58) cancer cells, transfected with siRNAs as indicated. nsi, non-targeting siRNA. [file 13046_2023_2785_MOESM1_ESM.pdf]

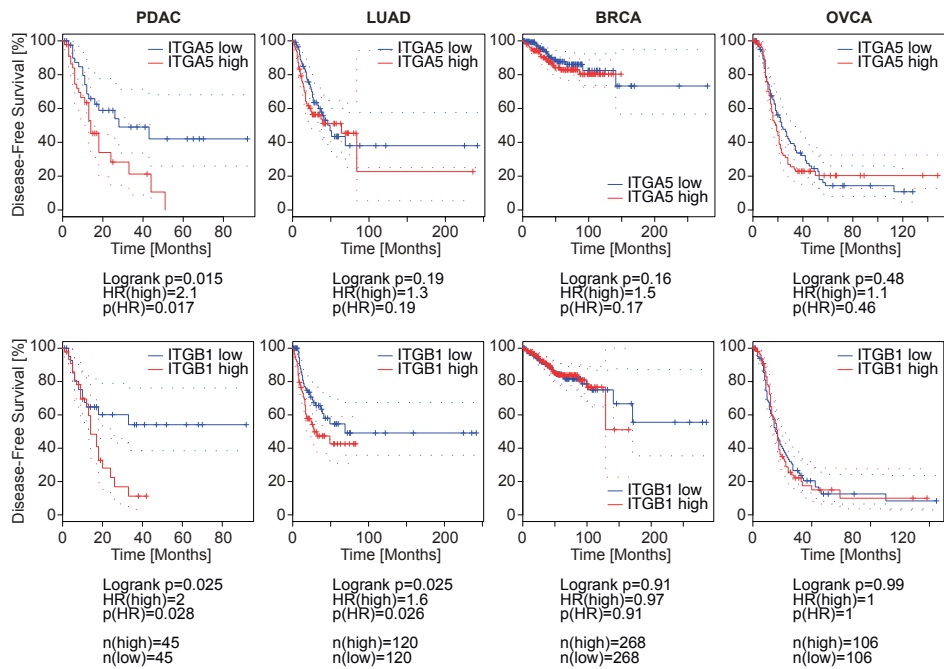

Supplement: Supplementary file 2 — Additional file 2: Supplemental Figure S2. Kaplan-Meier plots showing disease-free survival for patients with the indicated cancer types (PDAC, pancreatic ductal adenocarcinoma; LUAD, lung adenocarcinoma; BRCA, breast cancer; OVCA, ovarian carcinoma) stratified into high vs. low ITGA5 or ITGB1 mRNA expressing groups. Plots were generated and data statistically analyzed with GEPIA2 [46]. [file 13046_2023_2785_MOESM2_ESM.pdf]

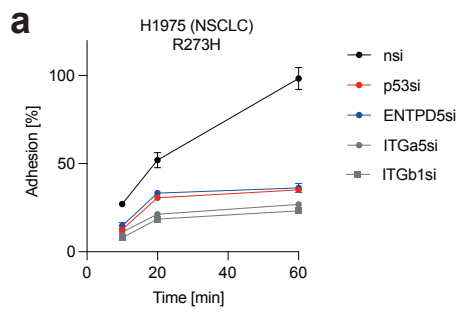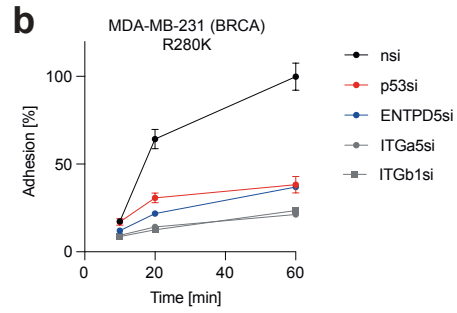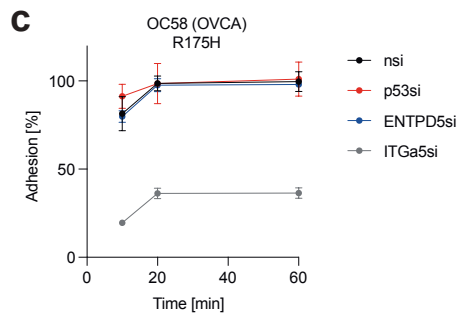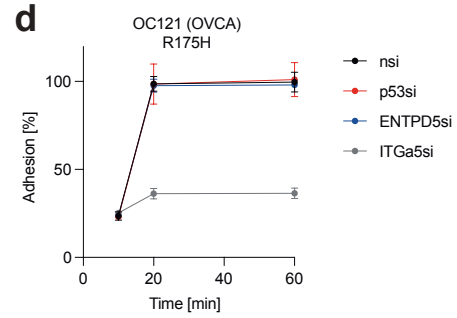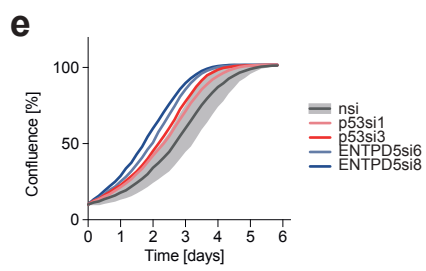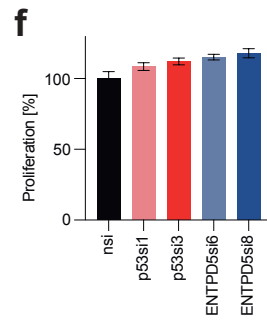

Supplement: Supplementary file 3 — Additional file 3: Supplemental Figure S3. Mutant p53 signaling via ENTPD5 is required for FN-mediated cell adhesion, migration, and invasion. a-d Adhesion kinetics of (a) H1975, (b) MDA-MB-231, (c) OC58 and (d) OC121 cells to fibronectin (FN) following siRNA-mediated depletion of p53, ENTPD5, ITGA5 or ITGB1. Adhesion is expressed as the percentage of the seeded cell number. e-f Proliferation effect of mutant p53 and ENTPD5. MIA PaCa-2 cells were transfected with siRNAs targeting p53 and ENTPD5 and analyzed by real-time live cell imaging. Non-targeting siRNA (nsi) is shown as control. e Confluence curves. Shown is the mean confluence of n=3 replicates. Shading for nsi-transfected cells indicates the SD. f Proliferation was quantified as the area under the confluence curve and normalized to nsi control cells as 100%. Shown is the mean ± SD (n=3 replicates) [file 13046_2023_2785_MOESM3_ESM.pdf]

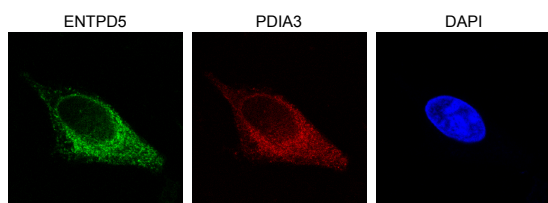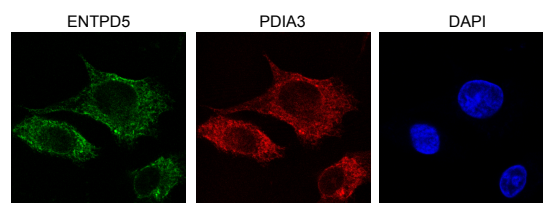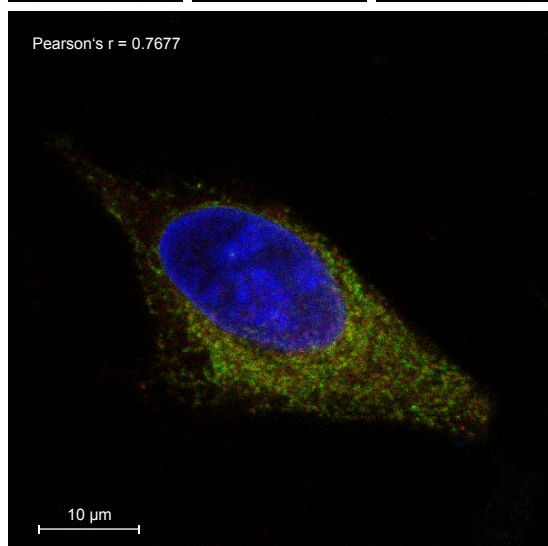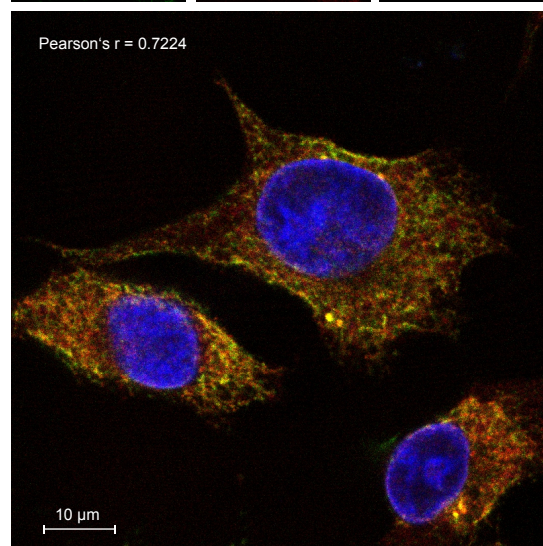

Supplement: Supplementary file 4 — Additional file 4: Supplemental Figure S4. ENTPD5 co-localizes with PDIA3 in the endoplasmic reticulum. Shown are representative confocal immunofluorescence microscopy images of MIA PaCa-2 cells stained with antibodies against the endoplasmic reticulum marker PDIA3 (protein disulfide isomerase family A member 3, also known as ERp57) and ENTPD5. Shown is the Pearson correlation coefficient for co-localization calculated with the Coloc 2 plug-in in ImageJ. [file 13046_2023_2785_MOESM4_ESM.pdf]

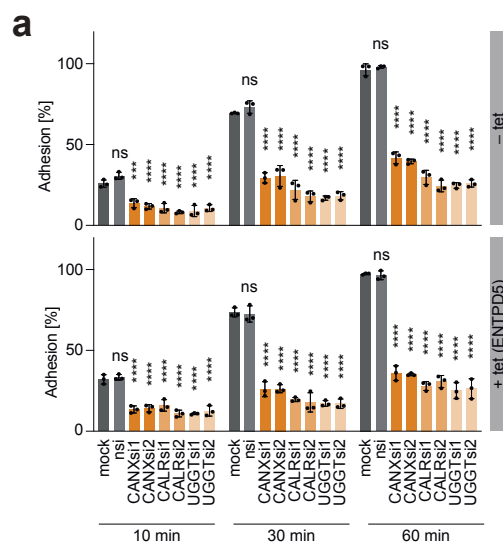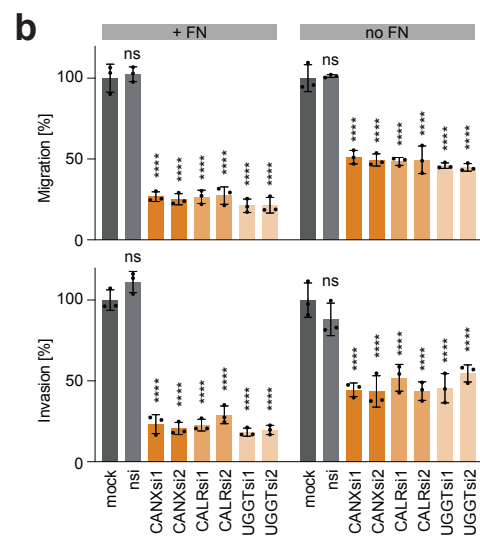

Supplement: Supplementary file 5 — Additional file 5: Supplemental Figure S5. CANX/CALR chaperones are essential for mutant p53-dependent ITGA5 activity. a FN-mediated adhesion of MIA PaCa-2 cells with tet-inducible expression of ENTPD5. Cells were treated with doxycycline 24 hours before transfection of indicated siRNAs and analyzed as in Fig. 2. b Migration and invasion of MIA PaCa-2 cells in the absence and presence of FN following depletion of CANX, CALR, and UGGT. All results are shown as mean ± SD (n=3 independent experiments). Statistical significance was tested using two-way ANOVA followed by Dunnett’s multiple comparisons test: ***, p<0.001; ****, p<0.0001; ns, not significant. Mock: non-transfected cells; nsi: non-targeting siRNA control. [file 13046_2023_2785_MOESM5_ESM.pdf]

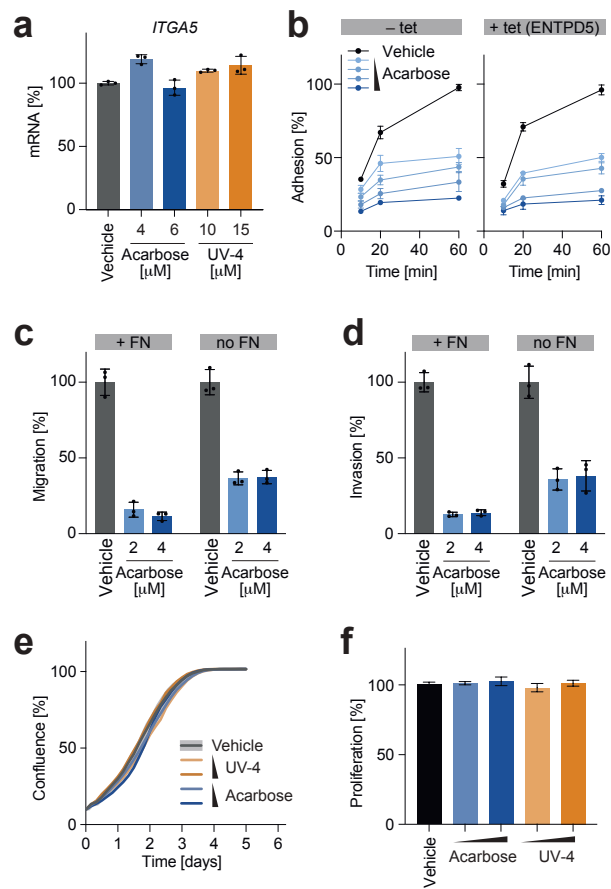

Supplement: Supplementary file 6 — Additional file 6: Supplemental Figure S6. α-glucosidase inhibitors block mutant p53-dependent ITGA5 expression and function. a RT-qPCR analysis of ITGA5 mRNA expression in MIA PaCa-2 cells treated with Acarbose and UV-4 as indicated. Shown is the GAPDH-normalized expression relative to vehicle-treated control cells as 100% (n=3 replicates). b Adhesion kinetics on FN of Acarbose-treated MIA PaCa-2 cells with tet-inducible ENTPD5 expression. c-d Migration and invasion of Acarbose-treated MIA PaCa-2 cells in the absence and presence of FN. e-f MIA PaCa-2 cells were treated with Acarbose or UV-4 and analyzed by real-time live cell imaging. e Confluence curves. Shown is the mean confluence of n=3 replicates. Shading for vehicle-treated control cells indicates the SD. b Proliferation was quantified as the area under the confluence curve and normalized to vehicle-treated control cells as 100%. All results are shown as mean ± SD (n=3 replicates). [file 13046_2023_2785_MOESM6_ESM.pdf]

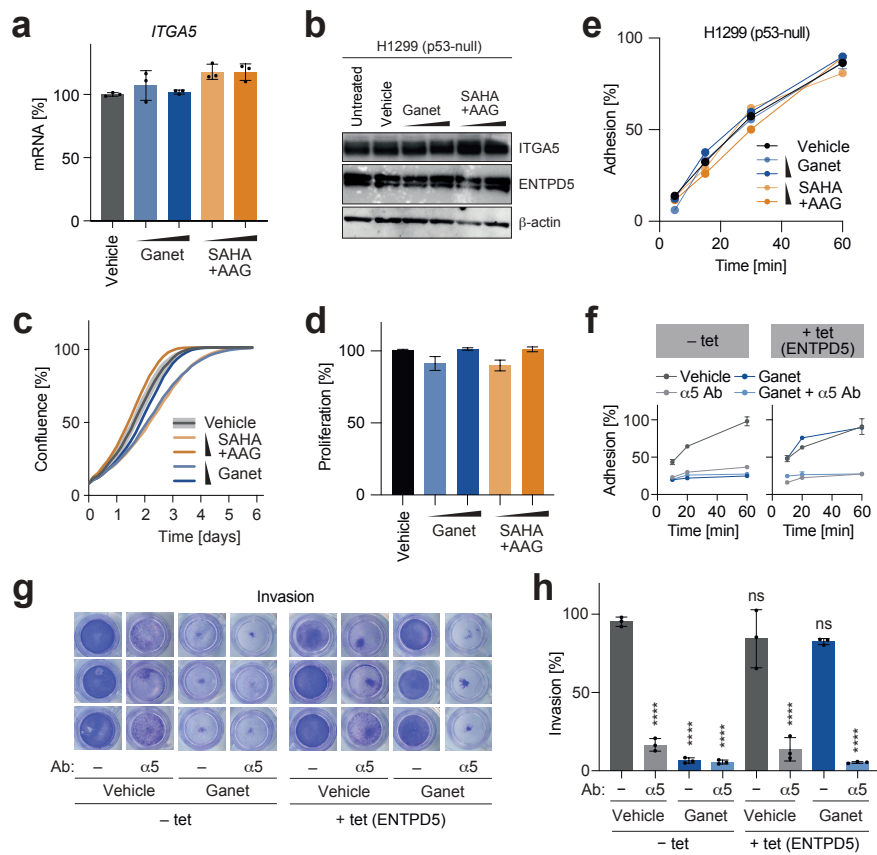

Supplement: Supplementary file 7 — Additional file 7: Supplemental Figure S7. Degradation of mutant p53 blocks ITGA5-mediated cancer cell motility. a RT-qPCR analysis of ITGA5 mRNA expression in MIA PaCa-2 cells treated with Ganet (0.5 or 1 μM) or SAHA+AAG (10 or 15 μM). Shown is the GAPDH-normalized expression relative to vehicle-treated control cells as 100% (n=3 replicates). b Western blot of p53-null H1299 cells for ITGA5 and ENTPD5 expression following treatment as in (a). c-d MIA PaCa-2 cells were treated as in (a) and analyzed by real-time live cell imaging. c Confluence curves. Shown is the mean confluence of n=3 replicates. Shading for vehicle-treated control cells indicates the SD. d Proliferation was quantified as the area under the confluence curve and normalized to vehicle-treated control cells as 100%. e Adhesion on FN of p53-null H1299 cells treated as in (a). f-h MIA PaCa-2 pIND-ENTPD5 cells (with tet-inducible expression of ENTPD5) were treated with doxycycline (+tet), Ganet (0.5 µM) and ITGA5-blocking antibody (α5) as indicated. f Adhesion on FN. g-h Invasion in the presence of FN. Statistical significance was tested using one-way ANOVA followed by Dunnett’s multiple comparisons test: ****, p<0.0001; ns, not significant. All results are shown as mean ± SD (n=3 replicates). [file 13046_2023_2785_MOESM7_ESM.pdf]
